# Hydrogel metapad with ultrasound transparency and broadband focusing for biomedical imaging

**DOI:** 10.1093/nsr/nwag048

**Published:** 2026-01-28

**Authors:** Jinhu Zhang, Deshuai Yu, Tianye Zhang, Chengtian Gao, Erqian Dong, Zhongchang Song, Chaoyu Fan, Zhehao Han, Fabrice Lemoult, Mathias Fink, Zhanxiang Wang, Youhui Lin, Yu Zhang

**Affiliations:** Key Laboratory of Underwater Acoustic Communication and Marine Information Technology of the Ministry of Education, College of Ocean and Earth Sciences, Xiamen University, Xiamen 361005, China; Institut Langevin, ESPCI Paris, PSL University, Centre national de la recherche scientifique (CNRS), Paris 75005, France; Department of Physics, Research Institute for Biomimetics and Soft Matter, Fujian Provincial Key Laboratory for Soft Functional Materials Research, Xiamen University, Xiamen 361005, China; Key Laboratory of Underwater Acoustic Communication and Marine Information Technology of the Ministry of Education, College of Ocean and Earth Sciences, Xiamen University, Xiamen 361005, China; Department of Neurosurgery and Department of Neuroscience, Fujian Key Laboratory of Brain Tumors Diagnosis and Precision Treatment, Xiamen Key Laboratory of Brain Center, the First Affiliated Hospital of Xiamen University, Xiamen 361005, China; School of Medicine, Xiamen University, Xiamen 361105, China; Key Laboratory of Underwater Acoustic Communication and Marine Information Technology of the Ministry of Education, College of Ocean and Earth Sciences, Xiamen University, Xiamen 361005, China; Key Laboratory of Underwater Acoustic Communication and Marine Information Technology of the Ministry of Education, College of Ocean and Earth Sciences, Xiamen University, Xiamen 361005, China; Department of Physics, Research Institute for Biomimetics and Soft Matter, Fujian Provincial Key Laboratory for Soft Functional Materials Research, Xiamen University, Xiamen 361005, China; Wenzhou Medical University, Wenzhou 325000, China; Institut Langevin, ESPCI Paris, PSL University, Centre national de la recherche scientifique (CNRS), Paris 75005, France; Institut Langevin, ESPCI Paris, PSL University, Centre national de la recherche scientifique (CNRS), Paris 75005, France; Department of Neurosurgery and Department of Neuroscience, Fujian Key Laboratory of Brain Tumors Diagnosis and Precision Treatment, Xiamen Key Laboratory of Brain Center, the First Affiliated Hospital of Xiamen University, Xiamen 361005, China; School of Medicine, Xiamen University, Xiamen 361105, China; Department of Physics, Research Institute for Biomimetics and Soft Matter, Fujian Provincial Key Laboratory for Soft Functional Materials Research, Xiamen University, Xiamen 361005, China; Key Laboratory of Underwater Acoustic Communication and Marine Information Technology of the Ministry of Education, College of Ocean and Earth Sciences, Xiamen University, Xiamen 361005, China; State Key Laboratory of Submarine Geoscience, School of Ocean and Civil Engineering, Shanghai Jiao Tong University, Shanghai 200240, China

**Keywords:** acoustic metamaterial, hydrogel, ultrasound transparency, broadband focusing, ultrasound imaging

## Abstract

Imparting broadband transparency and focusing on flexible ultrasound bioelectronics can significantly enhance the capabilities for precise evaluation of tissues and organs as well as treatment of diseases. While existing wearable ultrasound devices, whether rigid or stretchable, often merge rigid ultrasound element arrays with soft materials to achieve a reliable interface on the human skin, they lack controlled artificial microstructures, resulting in compromised ultrasound transparency and challenges in achieving broadband focusing. Here, we report a metapad made of hydrogel metamaterials with high ultrasound transparency and broadband focusing by controlling the hydrogel porosity at a sub-wavelength scale. The hydrogel metapad achieves near-perfect acoustic impedance matching with tissues, low attenuation loss, broadband transmission, and high focusing intensity gain. Ultrasound imaging simulations further show a significant improvement in imaging contrast near the focal region when using the hydrogel metapad. Practical applications demonstrate that it enhances the imaging capabilities of ultrasound probes for vital human organs, including blood vessels and the heart. Our hydrogel metapad holds great potential for advancing soft acoustic functional devices, capable of seamlessly interfacing with both biological tissues and aquatic environments.

## Teaser

A soft, biocompatible hydrogel metapad with a gradient porous structure achieves simultaneous high ultrasound transparency and broadband focusing, boosting echo signals from target regions and enhancing contrast in ultrasound imaging.

## INTRODUCTION

Wearable devices that can monitor human physiology with high quality, in a non-invasive and radiation-free manner, represent a significant trend in precision and digital medicine treatment [1–5]. Visualizing physiological signals from deep tissues and internal structures is an essential method for obtaining crucial information about health and disease [6]. Particularly, the accurate evaluation and treatment of tissue and organ functions depend on high-quality ultrasound focusing and imaging capabilities [7]. Moreover, ultrasound transparency allows a device to blend into the surrounding field with the advantage of high energy transmission [8–10]. As a result, an acoustically transparent and focusing device can advance healthcare systems [11], address significant questions in developmental biology [12], and play a vital role in the field of wave physics [13].

Conventional ultrasound focusing and imaging techniques typically rely on rigid high-density element arrays or curved lenses [14,15]. These methods often entail complex circuit modulation, extensive data processing and bulky acoustic systems, making them impractical for applications requiring compact and on-chip ultrasound devices. In contrast, emerging wearable ultrasound devices have shown the potential for long-term continuous imaging of diverse organs by integrating ultrasound element arrays into a soft elastic matrix that matches the skin [2,3,5,16–18]. To

ensure high diagnostic performance in ultrasound imaging, these devices primarily rely on coupling materials (liquid, gel-based, semi-dry, and dry) for transmitting ultrasound waves into the tissues [19]. Over the years, various liquid-/gel-based materials such as TM-100 (Jinya) and AQUASONIC 100 (Parker Labs) have been widely utilized as ultrasound couplants in biomedical ultrasound. Moreover, there has also been a growing interest in exploring hydrogel materials as coupling materials for ultrasonic imaging across a range of anatomical sites, including various organs [2,20], intraoral regions [21–23], and areas with high curvature [24]. Researchers have also developed semi-dry and dry couplants based on polymers (such as polypropylene, polyurethane, polyethylene, and elastomers) due to their stability and structural support [25,26]. Despite these advancements, conventional couplants suffer from limitations such as non-microstructural functional design, easily trapped air bubbles, acoustic impedance mismatch, and high energy attenuation [19,27,28] (Fig. S1, Tables S1 and S2).

**Figure 1.**
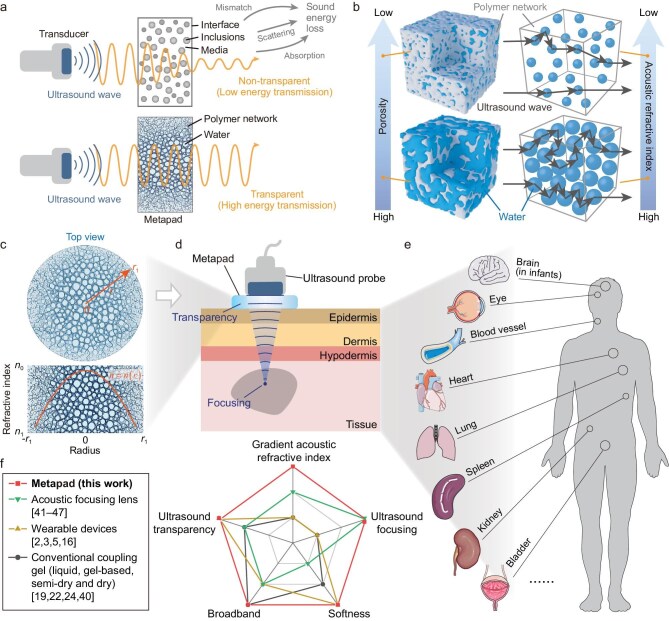
Design and physical characteristics of the metapad. (a) Schematic illustration of the difference in ultrasound energy-transmission efficiency between the metapad and other materials attributed to absorption, scattering, and impedance mismatch. (b) The metapad exhibits a tunable gradient acoustic refractive index, determined by its porosity due to multiple scattering of ultrasound waves. (c) Gradient acoustic refractive index of the metapad. (d) Schematic diagram of the metapad with both focusing and transparency functions for biomedical ultrasound. (e) Metapad-modulated ultrasound probes enable high-quality diagnosis and treatment of diverse human tissues and organs with enhanced contrast, accuracy, and cost-effectiveness. (f) Comparison of physical characteristics between existing ultrasound devices and the metapad [2,3,5,16,19,22,24,40–47].

Although metamaterial devices have successfully achieved innovative wave propagation properties [13,29–33] such as impedance matching, energy redirection and focusing, cloaking, super-resolution imaging, etc., challenges are still highlighted for their application in biomedical fields (Fig. 1a, Tables S1 and S3). Due to the absorption of matrix material, multiple scattering of macroscopic solid scatterers, and the attenuation caused by the impedance mismatch between the probe and tissues, current medical ultrasound performance of metamaterials is significantly weakened [34]. In addition, conventional gradient-index solid metamaterials cannot be applicable over an ultrasonic broadband range due to the size limitation of the macroscopic structure [35]. Previous studies have introduced the soft metagel, bioinspired hydrogel transformer, and soft meta-structure for ultrasound sensing [36–39]. However, the simultaneous achievement of ultrasound transparency and broadband focusing remains unexplored.

To address these challenges, we present a biocompatible metapad made of hydrogel metamaterials that uniquely integrates both ultrasound transparency and broadband focusing, which has not been achieved by previous ultrasound devices (Tables S1–S3). We exploit the scattering interaction between the hydrogel polymer network and water within microscopic pores to precisely control local acoustic properties (Fig. 1b). In cases where the design principle involves the precise control of the local statistical distribution (porosity) of subwavelength pores, the metapad can be regarded as a distinct class of acoustic metamaterials. This strategy generates a precisely controlled gradient acoustic refractive index (ranging from 0.48 to 0.98) allowing for precise ultrasound focusing at targeted focal points (Fig. 1c and d). Simultaneously, the metapad also demonstrates remarkable ultrasound transparency with an acoustic impedance (1.5–2.6 MRayl) matched to tissues and low acoustic attenuation (0.1–0.8 dB/cm at 2 MHz), facilitating full transmission and focusing across a wide frequency range in biological tissues. Acting simultaneously as a transparent coupling medium and a broadband focusing lens, the metapad significantly improves echo signal intensity from targeted regions, thereby enhancing contrast in ultrasound imaging (Fig. 1e). This breakthrough potentially opens up avenues for high-contrast, accurate, and cost-effective ultrasound diagnosis and treatment in clinical medicine (Fig. 1f).

## RESULTS

### Design strategy of the metapad with gradient acoustic refractive index

The metapad exhibits a gradient acoustic refractive index, denoted as *n*. The multiple scattering between the polymer network and water in the pores is responsible for the tunable gradient acoustic refractive index of hydrogel metamaterials. This scattering behavior and resulting effective parameters can be tuned profoundly through the porosity (Fig. 1b). Decreasing the porosity results in a corresponding decrease in the filling fraction of water as scatterers, which then reduces the acoustic refractive index.

**Figure 2.**
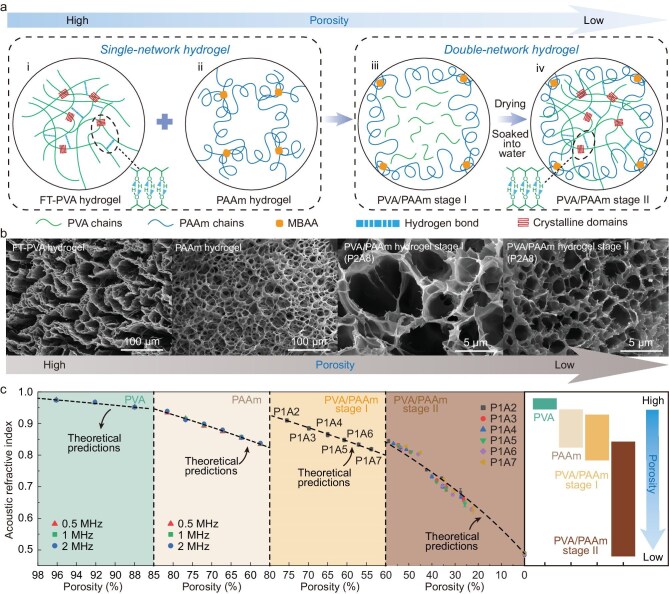
Hydrogel metamaterials with gradient acoustic refractive index manufactured by controlling its porosity at the subwavelength scale for the construction of the metapad. (a) Microscopic network design of the metapad with different acoustic refractive index based on four types of hydrogels: (ⅰ) FT-PVA hydrogel: physical cross-linking of PVA polymer networks via crystalline domains using a cyclic freezing–thawing (FT) method, yielding a high acoustic refractive index; (ⅱ) PAAm hydrogel: covalent cross-linking of polyacrylamide chains by *N,N*′-methylenebisacrylamide (MBAA; yellow squares), exhibiting a slightly lower acoustic refractive index than FT-PVA hydrogels; (ⅲ) PVA/PAAm stage I hydrogel: UV-initiated radical polymerization of AAm in the presence of PVA with a controlled PVA/AAm mass-fraction ratio, resulting in a reduced acoustic refractive index; (ⅳ) PVA/PAAm stage II hydrogel: drying and subsequent re-swelling of the PVA/PAAm stage I hydrogel, forming a double-network (DN) structure with an exceptionally low acoustic refractive index. (b) SEM images of the four types of hydrogel metamaterials mentioned above showing the gradient variation of porosity. (c) The acoustic refractive index of PVA, PAAm, and PVA/PAAm hydrogels as a function of porosity. The black dashed lines represent predictions from the theoretical model, and the symbols were experimentally measured. Error bars represent standard deviation of three independent samples (*n* = 3), and error bars smaller than the symbol size are omitted. The figure on the right shows the tunable range of the refractive index.

To demonstrate the universality of the multiple scattering behavior in hydrogels, two different types of hydrogels were selected for our investigation: physically cross-linked poly(vinyl alcohol) (PVA) and chemically cross-linked polyacrylamide (PAAm). The PVA solution underwent cyclic freezing-thawing, while the acrylamide (AAm) solution was UV-crosslinked to prepare hydrogels with a higher acoustic refractive index (Fig. 2a). However, achieving a low acoustic refractive index in single-network hydrogels may be challenging due to their low solubility or burst aggregation. To address this, we adopted a double-network strategy by constructing a hydrogel with low polymer porosity. Specifically, AAm monomer, cross-linker, and initiator were added to the PVA solution to form a pre-polymerized solution, which was then UV-crosslinked to produce composite hydrogels (referred to as PVA/PAAm stage I). PVA exerts a decelerating effect on the polymerization reaction of acrylamide and suppresses the burst polymerization of high-concentration AAm solutions, thereby further increasing the denseness of the polymer network in hydrogels. Following complete drying and re-swelling of the composite hydrogel, the hydroxyl groups arranged on the PVA chains form high-density hydrogen bonds, resulting in the formation of a PVA crystalline network, and yielding the double-network hydrogel (referred to as PVA/PAAm stage II) (Figs S2 and S3; Supplementary Discussion 1). Significantly, by closely packing the polymer chains and effectively controlling the degree of re-swelling in the final step, we achieved a substantial decrease in porosity, resulting in hydrogels with a lower acoustic refractive index. Scanning electron microscopy (SEM) images confirmed a decrease in porosity of the polymer network when transitioning from single-network to double-network hydrogels (Fig. 2b).

The acoustic refractive index (relative to water) of PVA, PAAm, and PVA/PAAm hydrogels of stage I and stage II were measured (Fig. 2c, Fig.S4). The acoustic refractive index of the hydrogel decreases with a gradual decrease in porosity. For single-network systems, the acoustic refractive index of PVA hydrogels was tuned in the range of 0.94 to 0.98, while that of PAAm hydrogels was tuned in the range of 0.82 to 0.94. In contrast, the design strategy of constructing double networks enables a significant decrease in the refractive index of hydrogels. Specifically, in the case of the double network hydrogel PVA/PAAm stage II obtained by drying and re-swelling, the acoustic refractive index of P1A7 and P1A6 (P*x*A*y* refers to the mass ratio of PVA to AAm as *x*:*y*) was significantly reduced to 0.627 at 22.3% porosity. Furthermore, the minimum acoustic refractive index can be achieved when the double network hydrogel is fully dried resulting in zero porosity. This drying and re-swelling approach is simple and easy to implement and is reversible. This process establishes a robust and repeatable correlation between porosity and the acoustic refractive index (Fig. S5). The acoustic properties at specific porosities remain consistent across multiple cycles and align with theoretical predictions, thereby ensuring predictable performance for practical applications. A large acoustic refractive index range (0.48 to 0.98) of the metapad is desirable for designing functional ultrasound devices with extreme acoustic properties. Nonetheless, it should be noted that the hydrogel’s ultrasound transparency and softness are most effective when hydrated. While fully dried hydrogels exhibit the lowest refractive index, they are also relatively stiff and have higher acoustic losses.

The modulation of the acoustic refractive index in the hydrogel metamaterials can be quantitatively understood based on a multiple scattering model; considering that the scatterers are randomly distributed in the surrounding medium. The behavior of the scattering medium is characterized by the complex propagation constant *k*. The effective wavenumber ${k}_{{\mathrm{eff}}}$ can be calculated within the framework of multiple scattering theory and can be written using the perturbation approach as [48]


(1) \begin{eqnarray*}
{k}_{{\mathrm{eff}}}^2 = {\left( {\frac{w}{{{c}_{{\mathrm{eff}}}}} + j{\alpha }_{{\mathrm{eff}}}} \right)}^2 = {k}_0^2 + {\delta }_1{\eta }_0 + {\delta }_2{\eta }_0^2 + \cdot \cdot \cdot ,
\end{eqnarray*}

where ${\eta }_0$ is the number of scatterers. Various multiple scattering theoretical models have been proposed, and it has been concluded that the effective wavenumber ${k}_{{\mathrm{eff}}}$ may be specified explicitly in terms of the number of scatterers per unit volume and the far-field amplitude $f(\theta )$ obtained for a single scatterer. In this context, we utilize the Waterman–Truell model [49] to obtain the effective wavenumber ${k}_{{\mathrm{eff}}}$, which is widely used in physics for concentrated suspensions [32]:


(2) \begin{eqnarray*}
{\left( {\frac{{{k}_{{\mathrm{eff}}}}}{{{k}_0}}} \right)}^2 = {\left[ {1 + \frac{{2\pi {\eta }_0f(0)}}{{{k}_0^2}}} \right]}^2 - {\left[ {\frac{{2\pi {\eta }_0f(\pi )}}{{{k}_0^2}}} \right]}^2,
\end{eqnarray*}

where $f(0)$ and $f(\pi )$ are the forward and backward scattering amplitudes for a single scatterer, respectively, which determine the coefficients ${\delta }_1$ and ${\delta }_2$. The single-scatterer amplitudes are given by


(3) \begin{eqnarray*}
\begin{array}{@{}l@{}} f(0) = \left( {{1 / {ik}}} \right)\sum\limits_{n = 0}^\infty {\left( {2n + 1} \right){B}_n}, \\ f(\pi ) = \left( {{1 / {ik}}} \right)\sum\limits_{n = 0}^\infty {{{\left( { - 1} \right)}}^n\left( {2n + 1} \right){B}_n}. \end{array}
\end{eqnarray*}

Here the polymer network is used as the matrix and the water in pores as scatterers. $f(0)$ and $f(\pi )$ simply depend on the material mechanical parameters of both the matrix (*ρ*_0_, *K*_0_, *G*_0_) and the scatterers (*ρ*_1_, *K*_1_, *G*_1_). According to the SEM images, the pores of the polymer networks in the metapad are on the micron level. The largest pores of our designed hydrogels do not exceed 50 μm, which is much smaller than the working wavelength *λ* ($= {{{c}_0} / f} = 0.742{\rm mm}$, $f = 2{\rm MHz} $). In such a long-wavelength regime, the first three expansion coefficients (*B*_0_, *B*_1_, *B*_2_) are significant. Therefore, the effective wavenumber ${k}_{{\mathrm{eff}}}$ is derived as [50,51]


(4) \begin{eqnarray*}
k_{{\mathrm{eff}}}^2 &=& k_0^2\left( {\frac{{\varphi {\rho }_1 + \left( {1 - \varphi } \right){\rho }_0}}{{{\rho }_0}}} \right)\\
&&\times \Bigg( 1 - \Bigg( \frac{{3 ( {{K}_1 - {K}_0})}}{{3{K}_1 + 4{G}_0}}\\
&&-\, \frac{{20{G}_0 ( {{G}_0 - {G}_1})}}{{{G}_0 ( {9{K}_0 + 8{G}_0} ) + 6{G}_1 ( {{K}_0 + 2{G}_0} )}} \Bigg)\varphi \Bigg),\\
\end{eqnarray*}

where $\varphi $ is the volume fraction of scatterers, indices 0 and 1 refer to the material parameters of the matrix and scatterers, respectively. We can then extract the effective sound speed and refractive index based on


(5) \begin{eqnarray*}
\begin{array}{@{}*{1}{l}@{}} {{c}_{{\mathrm{eff}}} = \displaystyle\frac{w}{{{k}_{{\mathrm{eff}}}}}},\\
{{n}_{{\mathrm{eff}}} = \displaystyle\frac{{{c}_{{\rm {water}}}}}{{{c}_{{\mathrm{eff}}}}}}. \end{array}
\end{eqnarray*}

Thus, the effective acoustic refractive index of the designed hydrogel metamaterials in Fig. 2c can be plotted as a function of the porosity. Taking PAAm hydrogel metamaterials for example, we consider water scatterers (*ρ*_1_ = 0.998 g/cm^3^, *K*_1_ = 2.19 GPa, and *G*_1_ = 0) and PAAm polymer network matrix (*ρ*_0_ = 1.1 g/cm^3^, *K*_0_ = 10 GPa, and *G*_0_ = 5 MPa).

The theoretical prediction was then compared with experimental measurements of the acoustic refractive index. We calculated the coefficient of determination (*R*^2^) between the experimental data and the theoretical predictions for four distinct hydrogel metamaterial formulations. The analysis yielded high *R*^2^ values (Table S4), confirming the validity of the multiple scattering model in capturing the essential acoustic behavior of the hydrogel metamaterials. Frequencies of 500 kHz, 1 MHz, and 2 MHz were examined for comparison. Mid-frequency ultrasound (0.1–1 MHz) is typically used for ultrasonic non-destructive testing and high-resolution SONAR, while higher frequencies (1–10 MHz) are commonly used for biomedical ultrasound applications, such as B-scan ultrasound imaging and high-intensity focused ultrasound (HIFU). Notably, the theoretical model reveals the intricate relationship between the mechanical properties and acoustic behavior of hydrogel metamaterials. Changes in a single mechanical modulus, such as stiffness, do not significantly impact the acoustic refractive index of the hydrogel (Fig. S6). For instance, increasing the number of freeze-thaw cycles in PVA/PAAm hydrogels (freezing-thawing based on stage I) enhances crystallinity and stiffness but does not substantially alter the acoustic refractive index. Similarly, modifying the cross-linker MBAA content in PAAm hydrogels shows negligible effect on the acoustic refractive index.

### Ultrasound transparency characteristics of the metapad

**Figure 3.**
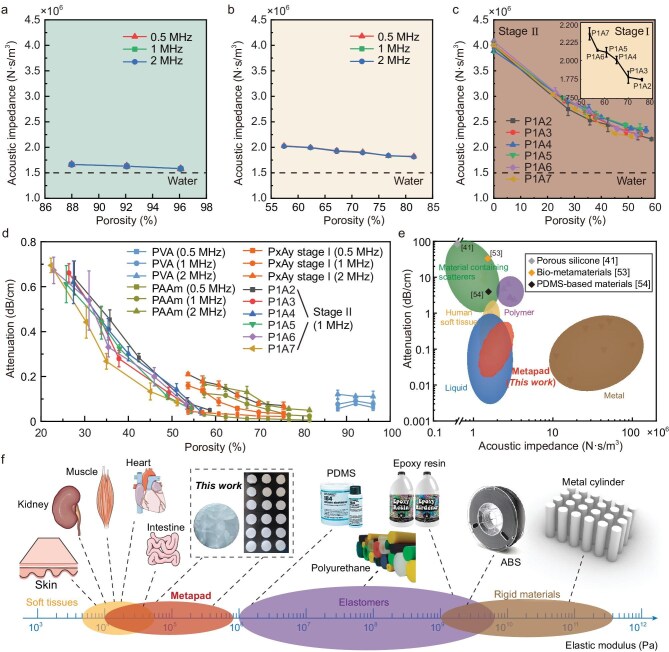
The metapad has high ultrasound transparency and low elastic modulus. (a–c) Acoustic impedance of the designed hydrogel metamaterials (PVA hydrogels, PAAm hydrogels, PVA/PAAm hydrogels of stage I and stage II) as a function of porosity. Error bars represent the standard deviation of three independent samples (*n* = 3), and error bars smaller than the symbol size are omitted. (d) Acoustic attenuation of the metapad at different porosities. (e) Comparison of acoustic attenuation (at 1 MHz) and impedance among the metapad and reported human soft tissues, liquid, materials containing scatterers, polymers, and metals. The plot shows that the acoustic properties of the metapad are exceptionally similar to those of water, skin, etc. The parameters of the same type of lens reported based on materials containing scatterers, such as porous silicone [41], bio-metamaterials [53], and PDMS-based materials [54] are marked with diamond shapes separately for comparison. (f) The elastic modulus of the metapad is close to that of soft tissues (such as skin, muscle, intestine, etc.), while other elastic and rigid materials commonly used in acoustic metamaterials have far higher moduli.

Ultrasound transparency is defined as the total transmission efficiency (${T}_{{\bf {\rm total}}} = T_{{\bf {\rm interface}}}^2 \times {e}^{ - 2\alpha h}$), which relies on both high transmission coefficient (${T}_{{\bf {\rm interface}}} = \frac{{4Z{Z}_{{\bf {\rm tissue}}}}}{{{{( {Z + {Z}_{{\bf {\rm tissue}}}} )}}^2}}$) at the interface and a negligible internal attenuation coefficient (*α*). To evaluate the ultrasound transparency of the metapad, we measured its acoustic impedance and attenuation coefficient. The acoustic impedance was obtained by *Z* = *ρc* (Fig. 3a–c). By increasing the porosity, the acoustic impedance of the metapad decreases; however, it typically remains below 4.07 × 10^6^ N·s/m^3^ (4.07 MRayl). Therefore, the acoustic impedance of the metapad remains within the same order of magnitude as that of water and tissue, enabling effective matching with these substances. Hydrogels, serving as the primary working material for acoustic matching devices, have found promising applications in underwater camouflaged actuators [9], bioinspired robots [8], and underwater hydrophones for listening [10], indicating the diverse potential uses of the metapad. Additionally, the acoustic attenuation of the metapad shows a dependence on the porosity (Fig. 3d). The P*x*A*y* hydrogels exhibit extremely low acoustic attenuation (<0.8 dB/cm) at ∼22.3% porosity, and the attenuation coefficient decreases rapidly after re-swelling. Acoustic impedance and attenuation coefficients of the four hydrogels for the subsequent metapad design at 0.5, 1, and 2 MHz are detailed in Table S5. We then performed a second-order polynomial regression analysis (*α* = *A* + *Bφ* + *Cφ*^2^) on the experimental data for the PVA/PAAm hydrogels (stage I and stage II). As shown in Fig. S7, the results yielded high coefficients of determination (*R*^2^ >0.99, Table S4), indicating a statistically significant deterministic correlation between attenuation and porosity. The absorption in polymers also depends on the acoustic frequency. The energy absorption of PVA and PAAm hydrogels under 1 MHz and 2 MHz ultrasound excitation is slightly higher than 0.5 MHz.

To further characterize the ultrasound transparency of the metapad in water environments, we compared the transmission loss and attenuation of hydrogel metamaterials with those of resin composite metamaterials and porous silicone rubber (Supplementary Discussion 2) [52]. The hydrogel metamaterials showed superior impedance matching and lower attenuation (Fig. 3e, Figs S8 and S9). The metapad can overcome the acoustic impedance mismatch in water-free polymers such as porous silicone rubber, polyurethane, epoxy resins, etc., as well as conventional materials such as metal and wood. In comparison to porous silicone rubber [41] and elastomers doped with metal powder [53,54], the metapad exhibits significantly lower acoustic attenuation, with a reduction of two orders of magnitude (Fig. 3e). Unlike air-filled porous materials, the low attenuation of the metapad composed of water and hydrophilic polymer chains can be attributed to the absence of the viscous and thermal boundary-layer losses described by Biot’s poroelastic theory [55] and the Johnson–Champoux–Allard model [56]. In water-saturated polymer networks, strong fluid–solid coupling suppresses the relative motion responsible for viscous dissipation in air-based porous media. Moreover, the near-incompressibility of water prevents pressure-induced temperature variations, effectively eliminating thermal conduction losses typical of gas-filled pores. In addition, we also compared the metapad with common coupling media, including various structured hydrogel pads, elastomer couplants, and acrylic tape, in terms of sound transmission efficiency and focusing efficiency (Table S2). The advantage of ultrasound transparency allows the metapad to be used as a promising coupling layer for ultrasound transmission with enhanced focusing capabilities.

The metapad demonstrates exceptional compliance, flexibility, and softness, allowing for seamless adaptation to external unstructured environments. Quantitative compression testing of all metapad components, including pure PVA, PAAm, and PVA/PAAm hydrogels, confirms that their elastic moduli lie within the range of 10^4^ to 10^6^ Pa (Figs S10 and S11, Supplementary Discussion 3). Therefore, the metapad can function as a mechanically friendly interface, effectively connecting rigid ultrasound transducers to water or body tissues. With a low elastic modulus similar to that of biological soft tissues, the metapad’s elastic modulus is significantly lower, by several orders of magnitude, than that of metals, resins, acrylonitrile butadiene styrene (ABS), and other rigid materials typically used in metamaterials (Fig. 3f). This softness minimizes energy loss caused by the conversion and coupling of transverse and longitudinal waves during ultrasound propagation. The metapad’s exceptional compliance is highly advantageous for clinical applications, particularly on non-flat skin surfaces, as it ensures a reliable interface between human skin and ultrasound probes. Additionally, to ensure structural integrity under clinical probe compression, we evaluated the fatigue resistance of the four hydrogels used in the metapad design using cyclic loading tests. A maximum strain of 20% was applied to mimic the typical pressure exerted on human skin during examination. Over 100 continuous loading-unloading cycles, the stress-strain curves exhibited linear elasticity with negligible hysteresis or Mullins effect (Fig. S12). This indicates that the metapad could retain its dimensional integrity and mechanical resilience under repeated clinical handling.

### Broadband focusing characteristics of the metapad

**Figure 4.**
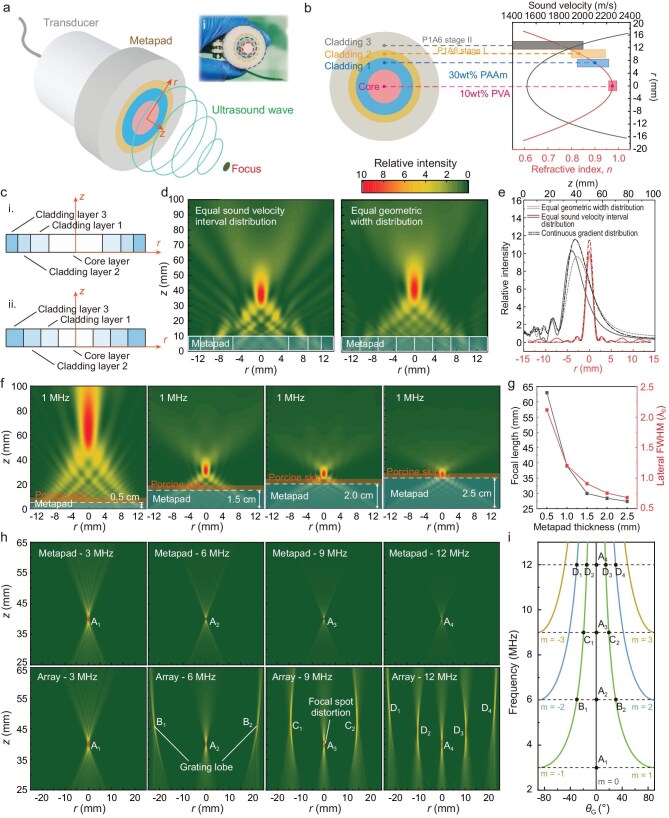
Ultrasound broadband focusing via the hydrogel metapad. (a) Schematic illustration of ultrasound wave propagation through the metapad to achieve focusing. Inset (i): radial acoustic refractive index distribution of the hydrogel metapad for broadband focusing. (b) The left image shows the top view of the designed metapad, and the right image shows its discretized index distribution within the metapad. Four color blocks represent the refractive index ranges of four types of hydrogel metamaterials, from which the metapad is fabricated with different acoustic refractive indices. (c) Schematic diagram of discretizing the planar metapad into a certain number of layers based on the distribution of (i) equal intervals of sound velocity and (ii) equal geometric width. (d) Acoustic intensity field patterns simulated at 1 MHz in the r-z planes with the two metapads, which are discretized by equal geometric width distribution and equal sound velocity interval distribution, respectively. (e) Comparative simulation of focal intensity profiles for different refractive index distributions. (f) Tunable ultrasound focusing capability of the hydrogel metapad. Simulated acoustic intensity distribution for the cases of the metapad with thicknesses of 0.5 cm, 1.5 cm, 2.0 cm, and 2.5 cm. The operating frequency is 1 MHz. (g) Dependence of acoustic focal length and lateral FWHM on the metapad thickness. (h) Simulated ultrasound intensity distributions at different frequencies for acoustic focusing by the gradient hydrogel metapad and a conventional array. (i) The theoretical relationship between operating frequency and the spatial angle (*θ*_G_) for the main lobe (*m* = 0) and the first three orders of grating lobes (*m* = ±1, ±2, ±3). The labeled points (A, B, C, D) denote the spatial angles of the main lobe and side lobes that appear at different operating frequencies, as determined by Equation (10), and are consistent with the simulated results shown in (h).

To evaluate the focusing performance, we applied the hydrogel pad with a radial acoustic refractive index distribution (Fig. 4a and b). The thickness of the metapad was set to 1 cm. The refractive index profile was obtained by using the following coordinate transformation (Supplementary Discussion 4 and Fig. S13):


(6) \begin{eqnarray*}
\begin{array}{@{}*{1}{l}@{}}
{n\!\left( r \right) = {n}_0{\mathop{\mathrm{\ sech}}\nolimits} [ {g ( {\alpha r} )} ],}\\
{g\!\left( \xi \right) = {\xi / {\left( {1 + {\beta }_1{\xi }^2 + {\beta }_2{\xi }^4} \right).}}} \end{array}
\end{eqnarray*}

The focusing performance of the gradient hydrogel pad is well described by an analytical model based on ray theory (Supplementary Discussion 5). This model is based on the wave equation for a non-uniform medium:


(7) \begin{eqnarray*}
{\nabla }^2p\left( {{\boldsymbol{r}},w} \right) &-& \nabla \ln \rho \left( {\boldsymbol{r}} \right) \cdot \nabla p\left( {{\boldsymbol{r}},w} \right)\\
&&+ {k}^2p\left( {{\boldsymbol{r}},w} \right) = 0.
\end{eqnarray*}

Under the high-frequency approximation, the Eikonal equation can be derived as


(8) \begin{eqnarray*}
\frac{{\mathrm{d}}}{{{\mathrm{d}}s}}\left[ {n\left( {\boldsymbol{r}} \right)\frac{{{\mathrm{d}}{\boldsymbol{r}}}}{{{\mathrm{d}}s}}} \right] = \nabla n\left( {\boldsymbol{r}} \right).
\end{eqnarray*}

Solving Equation (8) yields the ultrasound ray trajectory (Fig. S13):


(9) \begin{eqnarray*}
r\left( z \right) = \frac{1}{\alpha }{\sinh }^{ - 1}\left[ {\sinh \left( {\alpha {r}_0} \right)\cos \left( {\alpha z} \right)} \right].
\end{eqnarray*}

For small values of $\alpha {r}_0$, Equation (9) simplifies to $r( z ) = {r}_0\cos ( {\alpha z} )$. Numerical simulations confirm that the hydrogel pad enables ultrasound focusing with high-energy and low aberration (Supplementary Discussion 6 and Fig. S14). This capability arises from the above modified hyperbolic secant profile and near-perfect acoustic energy transmission.

The discretization of the metapad employs a nested structure (Fig. S15). As shown in Fig. 4a and b, the metapad consists of three layers of concentric rings (cladding layers) and a rounded central part (core layer). The metapad’s radial acoustic refractive index was discretized using the equal sound velocity interval method (Fig. 4c) and compared with a scheme based on equal geometric widths. The equal sound-velocity interval scheme exhibits superior focusing quality (Fig. 4d). Specifically, it achieves a focal spot size (full width at half maximum—FWHM) of 1.701 mm, which is notably narrower than the 1.950 mm FWHM observed in the equal geometric width distribution method (Fig. 4e). Furthermore, the acoustic energy concentration at the focus is enhanced, with the equal sound velocity interval distribution yielding a 0.3 dB increase in peak intensity compared to the equal geometric width distribution. We further compared the focal profiles of the fabricated discrete structure with an ideal continuous gradient model. Although a slight reduction in peak intensity is observed due to the stepwise phase approximation, the discrete design maintains high focusing quality with measured lateral and axial FWHMs of 1.195*λ*_0_ and 15.941*λ*_0_, respectively. These values closely match the ideal continuous model (Fig. S14). Furthermore, the peak side lobe level (PSLL) of the discrete structure is −11.96 dB, comparable to the −12.52 dB of the ideal model. These results confirm that the quantization errors caused by the layered manufacturing process are negligible and do not result in significant focal broadening. However, it is anticipated that regardless of the discretization method, as the precision of the discretization increases and approaches the ideal gradient of sound velocity, the focusing effect will tend to converge.

To investigate the impact of metapad thickness on focal length and lateral FWHM, we conducted simulations by varying the metapad thickness from 0.5 cm to 2.5 cm (Fig. 4f). The corresponding focal length reduced from 62.9 mm to 27.4 mm, and the resolution FWHM narrowed from 2.12*λ*_0_ to 0.68*λ*_0_ (Fig. 4g). An explanation through theoretical derivation is detailed in Supplementary Discussion 5. Notably, the materials and dimensions of the metapad can be readily adjusted, specifically to tune focal lengths of ultrasound waves for meeting the requirements of distinct applications, including tissue and organ imaging and treatment at varying depths. Furthermore, the metapad demonstrates adaptability to curved tissue surfaces, as validated in tests on three different curvature tissue surfaces. Remarkably, the deformed metapad retained its ability for ultrasound focusing (Fig. S16).

To test the broadband characteristics of the metapad for ultrasound focusing, we compared the acoustic intensity distributions at various frequencies. Ultrasound focusing appears in the center of acoustic fields. The FWHM reduces from 8.85 mm (1.194λ_0_) at 0.2 MHz to 0.160 mm (1.290*λ*_0_) at 12 MHz (Fig. S17). Broadband focusing of the metapad facilitates the acquisition of maximum tissue information for enhanced contrast resolution and the ability to differentiate or resolve structures in the depth domain in medical ultrasound [57,58]. Furthermore, in contrast to conventional array-based systems that rely on hardware-defined element spacing, the metapad employs a fundamentally different strategy for ultrasound focusing. Conventional arrays with wavelength-scale elements are prone to spatial aliasing at high frequencies, leading to focal spot distortion or grating lobes [59] (Supplementary Discussion 7). The direction of these grating lobes (${\theta }_{\rm G}$) can be described by


(10) \begin{eqnarray*}
\sin {\theta }_{\rm G} \approx \sin {\theta }_{\rm F} - m\frac{\lambda }{d},
\end{eqnarray*}

where $\lambda $ is the wavelength, *d* is the array element spacing, ${\theta }_{\rm F}$ is the direction of the designed focus, and *m* is the grating lobe order. When the element spacing becomes large (e.g. $d > \lambda $), it is easy to find a non-zero integer *m* that satisfies this condition, resulting in real-valued ${\theta }_{\rm G}$. This gives rise to one or more grating lobes, which divert sound energy away from the intended focal region and distort the ideal focus point.

To illustrate this effect, simulations were performed at operating frequencies of 3, 6, 9, and 12 MHz. Given the array pitch of *d* = 0.513 mm, the critical diffraction limit defined by *d*/*λ* = 1 is reached at 3 MHz. Figure 4h presents the simulated acoustic intensity distributions for the gradient hydrogel metapad (top row) and a conventional phased array (bottom row). While both systems achieve effective focusing at 3 MHz (A_1_), the conventional array exhibits increasing degradation in performance at higher frequencies ($d > \lambda $) due to the emergence of grating lobes (B_1_, B_2_ at 6 MHz; C_1_, C_2_ at 9 MHz; and D_1_–D_4_ at 12 MHz), accompanied by focal spot distortion. These artifacts arise from spatial aliasing due to the array’s fixed element pitch, which becomes more pronounced at higher frequencies. These results directly reflect the spatial aliasing effects introduced by fixed element spacing in the array. In contrast, the gradient hydrogel metapad maintains a sharply confined and undistorted focal spot (A_1_–A_4_) across the entire frequency range, highlighting its broadband focusing capability. Figure 4i presents the theoretical relationship between operating frequency and the spatial angle (${\theta }_{\rm G}$) for the main lobe (*m* = 0) and the first three orders of grating lobes (*m* = ±1, ±2, ±3), as described by Equation (10). The labeled points A–D mark the spatial angles of the main and side lobes at various frequencies, showing excellent agreement with the simulated fields in Fig. 4h. As the frequency increases, the number of side lobes gradually increases; meanwhile, these side lobes approach closer to the main lobe, and the side-lobe level also gradually increases. For instance, at 12 MHz (*d*/*λ* = 4), our analysis confirms that the first-order grating lobes (D_2_ and D_3_, m = ±1) appear at ∼ ±14.48°, with the side-lobe level reaching as high as −6.27 dB. In contrast, the gradient metapad avoids such frequency-dependent angular dispersion because *d* is much smaller than the wavelength $\lambda $ (as the element spacing can be considered infinitesimally small in the gradient structure), thus ensuring consistent focusing across a wide frequency range.

**Figure 5.**
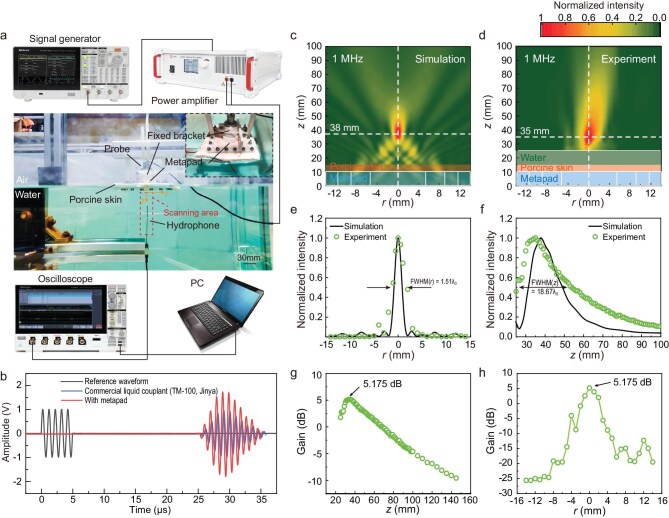
Experimental validation of ultrasound focusing via the hydrogel metapad. (a) Experimental setup for ultrasound focusing through porcine skin. The fixed bracket holds the probe and metapad and keeps them aligned in the center. (b) Ultrasound signals travel through the porcine skin with the hydrogel metapad or conventional commercial liquid couplant (TM-100, Jinya) from the transducer to the hydrophone with a source frequency of 1 MHz. (c and d) Simulated and experimental ultrasound intensity distribution of 1 MHz ultrasound waves excited by the probe through porcine skin after metapad manipulation. (e and f) Measured (green circles) and simulated (black line) normalized amplitude field distributions along the *r*-axis at *z* = 38 mm or *z* = 35 mm (e) and the *z*-axis for *r* = 0 mm (f). The FWHM are 1.51*λ*_0_ and 18.67*λ*_0_ along the *r*-axis and *z*-axis, respectively. (g and h) Experimental sound energy gain (dB) of ultrasound waves at 1 MHz after metapad manipulation through porcine skin. The gain through the focal point along the *z*-axis is shown in (g), and the gain through the focal point along the *r*-axis is shown in (h).

We also conducted experimental examinations to assess the feasibility of the metapad for focusing ultrasound waves through soft tissues. Porcine skin was utilized as the experimental sample due to its similarity to human skin [60]. The porcine skin was immobilized in a water tank, and the metapad was positioned between the ultrasound probe and the porcine skin (Fig. 5a). The ultrasound waves generated by the probe propagated through the porcine skin via the hydrogel metapad, resulting in a significantly higher amplitude of the ultrasound signal at the focus compared to a conventional commercial liquid couplant (TM-100, Jinya) (Fig. 5b). Focal peak negative pressure (PNP) measured via a needle hydrophone (ZS-1000) was ∼0.36 MPa at 1 MHz, yielding a Mechanical Index (MI) of 0.36. This value falls below the cavitation threshold (typically >0.7), confirming linear acoustic operation with negligible nonlinear risks. The derived Spatial-Peak Pulse-Average intensity (*I*_SPPA_) is ∼4.3 W/cm^2^, which is well below the Food and Drug Administration (FDA) limit of 190 W/cm^2^. Given the low duty cycle of standard diagnostic imaging (<1%), the Spatial-Peak Temporal-Average intensity (*I*_SPTA_) is estimated to be <43 mW/cm^2^, which remains orders of magnitude below the regulatory thermal limit (720 mW/cm^2^). Next, the ultrasound field was scanned and a distinct focal spot was observed at a focal depth of 38 mm in the simulation and 35 mm in the experiment, respectively (Fig. 5c and d). Figure 5e and f depicts the acoustic intensity distributions along both the transverse and axial directions at the focal spot. Specifically, the metapad’s focusing feature resulted in a notable energy gain (5.175 dB) at the focal spot relative to the same point with a conventional commercial liquid couplant (TM-100, Jinya) (Fig. 5g and h). This gain is defined by the formula *Gain =* 20lg(*P*_metapad_*_/_P*_TM-100_), where *P*_metapad_ and *P*_TM-100_ represent the sound pressure with the hydrogel metapad and conventional liquid couplant, respectively. Minor discrepancies between the experimental and simulated fields, such as focal broadening and axial shift, could be primarily attributed to phase aberrations induced by unavoidable thickness variations in the re-swollen PVA/PAAm stage II hydrogel layer, as well as additional scattering from the rigid mechanical holder not present in the idealized simulation.

### Enhancing plane-wave ultrasound imaging with the hydrogel metapad

To evaluate the impact of the hydrogel metapad on ultrasound imaging quality, we conducted simulations of plane-wave imaging in the azimuthal plane with and without the metapad using Field II. Plane-wave imaging represents a transformative shift in the medical ultrasound paradigm. In our simulations, the metapad was positioned between the transducer and the artificial phantom (Fig. S18a). The acoustic refractive index manifests as a delay of the signal in the time domain. Without the metapad, the emitted signals from each element have zero delay (black line in Fig. S18b). When the metapad is applied, relative delays are introduced between the emitted signals from different elements due to the refractive modulation of the metapad (red line in Fig. S18b). The refractive index profile was configured as specified in Equation (6). The results demonstrated that the hydrogel metapad effectively focused the plane waves emitted by the linear transducer array, yielding a sound energy gain exceeding 15 dB at the focal point (Fig. S18c).

**Figure 6.**
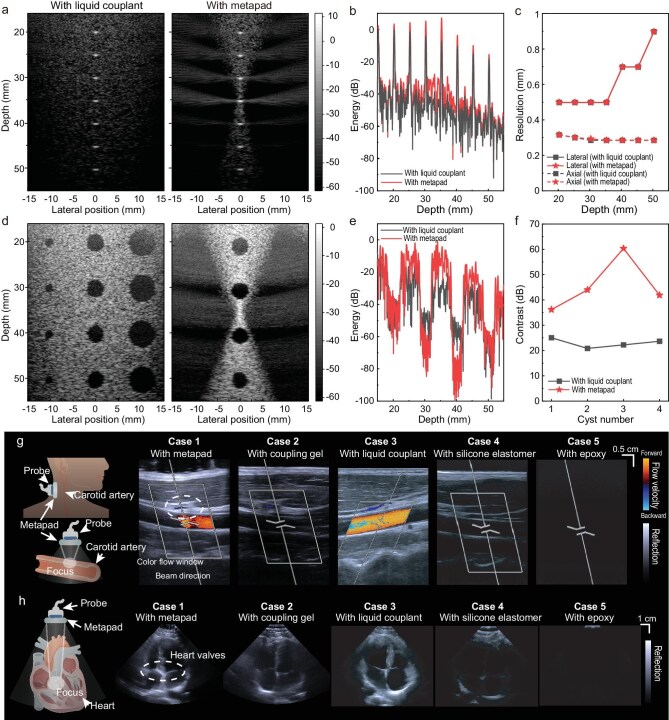
Ultrasound imaging via the hydrogel metapad. (a) Comparison of simulated PSF for some point object with the hydrogel metapad or common liquid couplant. The color scale is in decibels. (b) Energy as a function of depth at lateral position *x* = 0 obtained from PSF phantom imaging. (c) −10 dB lateral and axial resolution curves. (d) Comparison of plane wave imaging of anechoic cysts embedded in a tissue mimicking a phantom with the hydrogel metapad or common liquid couplant. The color scale is in decibels. (e) Energy as a function of depth at lateral position *x* = 0 obtained from cyst-phantom imaging. (f) Contrast of four cysts from superficial to deep at lateral position *x* = 0. (g) Schematics of the metapad attached to the neck of the subject to image the carotid artery. Comparison of color flow imaging of the carotid artery with metapad, coupling gel (pure PAAm hydrogel), commercial liquid couplant (TM-100, Jinya), silicone elastomer (Ecoflex 00–30), and epoxy (thermoset). The metapad significantly enhances the imaging quality of the focus region (white dashed lines). (h) Comparison of heart imaging with the metapad and other coupling materials.

The usual criteria for evaluating an imaging method include resolution and contrast. First, we analyzed the point spread function (PSF) for both imaging configurations. For point scatterers within the phantom, the PSFs obtained with and without the hydrogel metapad (using a conventional liquid couplant in the latter case) are shown in Fig. 6a. The localization of ultrasound energy induced by the metapad results in stronger reflections from these point targets, appearing as brighter features in the grayscale images. While tissue naturally attenuates sound waves, the metapad’s modulation significantly enhances the echo energy of these point targets, particularly near the focal region (Fig. 6b). Figure 6c shows that the −10 dB lateral and axial resolutions derived from the PSF remain unaffected by the presence of the hydrogel metapad. Furthermore, consistent with diffraction physics, the resolution naturally improves as the frequency increases from 3 MHz to 7.5 MHz (Fig. S19a–c). Crucially, the close agreement between the metapad and the control group confirms that the intrinsic resolution is preserved across a broad operating frequency range, ensuring that the signal enhancement does not come at the cost of image fidelity. In demonstrations of common carotid artery ultrasound imaging, the luminal diameter (LD) and intima-media thickness (IMT) measured using the metapad were consistent with those obtained using conventional liquid couplants (Fig. S20, Supplementary Discussion 8). This result further supports the reliability of the metapad in maintaining axial resolution in practical ultrasound imaging applications.

The performance of the hydrogel metapad was also assessed in terms of contrast. Contrast reflects the imaging method’s ability to detect anechoic objects, such as cysts, embedded in a homogeneous scattering medium. Figure 6d illustrates plane-wave imaging of anechoic cysts. It is evident that the hydrogel metapad effectively focuses ultrasound energy. Although this may limit the imaging field of view, it enhances the echo from the surrounding medium near the focal region while further reducing energy within anechoic regions (Fig. 6e). Near the focal location, the contrast level peaks at 60.33 dB—an ∼38 dB improvement compared to the 22.23 dB contrast of conventional plane-wave imaging with a common liquid couplant (Fig. 6f). A significant contrast improvement can also be consistently observed near the focal region over a broad frequency range (Fig. S19d–f). Therefore, we could predict that the metapad can effectively enhance contrast, particularly for imaging deep tissues with higher attenuation. In addition, we describe a quantitative approach to evaluating the effective imaging area for metapad-enhanced ultrasound imaging (Fig. S21; Supplementary Discussion 9).

We finally demonstrate three applications of the hydrogel metapad to enhance ultrasound imaging of human organs. In Fig. 6g, we present the imaging results of the carotid artery obtained with an ultrasound probe (10L4, ACUSON Sequoia) using the metapad. Parameters of the ultrasound probe and the discretized metapad for imaging are detailed in Tables S6 and S7. Compared with the normal coupling gel (pure PAAm hydrogels) without gradient refractive index modifications, the metapad focuses the ultrasound waves on the carotid artery region (white dashed lines) more clearly by significantly enhancing the echo. The metapad imaging results further provide continuous blood flow velocity data of the carotid artery with greater clarity than the normal coupling gel (Fig. S22). We clearly observed that the subject’s slightly faster blood flow velocity was ∼100 cm/s, which may be due to the subject lying down immediately for the test after the activity. The blood flow velocity after several minutes of resting was ∼80 cm/s, obtained using the normal coupling gels, and exhibited poor clarity. Figure 6h illustrates the imaging results of the heart obtained with the ultrasound probe (4V1, ACUSON Sequoia) using the metapad attached to the subject’s left side of the chest. The metapad’s acoustic transparency and focusing capabilities enable an image of the heart valves with greater contrast, as indicated by the white dashed lines. The metapad provides a more visible image of the four cardiac chambers due to a sharper contrast with the surrounding tissue. In contrast, the ultrasound imaging quality achieved with the normal coupling gel is notably inferior. A quantitative analysis comparing the performance of the metapad against the coupling gel and liquid couplant are detailed in Supplementary Discussion 10 (Fig. S23).

As depicted in Fig. 6g and h, both the metapad and commercial liquid couplant (TM-100, Jinya) are effective in clearly imaging the carotid arteries and the heart, outperforming other coupling materials. However, the metapad exhibits a unique capability in efficiently focusing acoustic energy on specific human organ regions (indicated by white dashed lines), a feature not observed with the liquid couplant. While liquid couplants are widely used and provide clear imaging as expected by clinicians, they lack the metapad’s ability to enhance ultrasound focusing. This makes the metapad more suitable for specific regions of interest. Beyond the cardiovascular system represented by blood vessels and the heart, the metapad could provide high-precision ultrasound focusing and imaging of muscles, digestive and respiratory systems, including biceps, stomach, lungs, and more, which will be tested in future studies (Fig. 1e). The metapad, featuring a gradient acoustic refractive index, presents a promising concept for future medical ultrasound devices that offer the combined functions of ultrasound focusing and transparency.

Notably, the biocompatibility of the metapad was systematically evaluated through a series of *in vitro* and *ex vivo* assays. Cytotoxicity was first assessed using a Cell Counting Kit-8 (CCK-8) assay, where comparable optical density (OD) values were obtained for cells cultured with or without extracts from the four types of hydrogels (Fig. S24a). The viability of MC3T3-E1 cells remained above 90% after 3 d of incubation, regardless of the different extracts, indicating negligible cytotoxicity (Fig. S24b). Fluorescent staining of MC3T3-E1 cells cultured on various hydrogels for 3 d revealed that the designed hydrogels have minimal impact on cell proliferation (Fig. S24c). Beyond cytocompatibility, the biological safety of the hydrogels was further examined in terms of their blood and skin compatibility. All samples exhibited hemolysis rates below 5% at body temperature after prolonged incubation (Fig. S25a). Moreover, given that the hydrogel is designed for use as an external ultrasound pad, its local skin compatibility was specifically evaluated. No noticeable erythema or swelling was observed after 6 h of direct skin contact, demonstrating good skin tolerance (Fig. S25b). Therefore, the metapad demonstrates excellent biocompatibility and biosafety, holding potential for biomedical ultrasound applications *in vitro* or *in vivo*.

## DISCUSSION

In summary, this work introduces a novel material framework in the field of soft acoustic metamaterials, with the potential to significantly advance ultrasound imaging and acoustic physics. By leveraging the multiple scattering of polymer networks with water in microscopic pores, we have demonstrated the theoretical prediction and experimental verification of a gradient acoustic refractive index for the first time. Moreover, the hydrogel’s high-water content facilitates remarkable ultrasound transparency. The resulting constructed metapad exhibits ultrasound transparency, broadband focusing, and soft characteristics. Designed as a novel ultrasound transparency and broadband focusing scheme, our metapad offers superior ultrasound imaging capabilities and continuous blood flow velocity data, making it a valuable tool in clinical ultrasound diagnosis and organ disease management.

High-quality ultrasound imaging capabilities, including ultrasound transparency, low energy attenuation, impedance matching, and broadband focusing, play a critical role in medical devices. Existing acoustic lens [27,41–45] and wearable ultrasound devices [2,3,16,22,24,40] lack one or more of the ideal properties required (Tables S1 and S3). In addition, complex circuit modulation, non-microstructural functional design, and non-conformal mismatched contacts inevitably hinder their practical utility. To contextualize our contribution within the current technological landscape, we performed a systematic head-to-head comparison with established ultrasound technologies, including commercial active linear arrays and emerging flexible/stretchable arrays (Table S8). While these existing systems rely on macroscopic discrete elements with a fixed pitch to manipulate sound, they inherently suffer from physical limitations such as spatial aliasing (grating lobes) when operating above a frequency threshold dictated by the element spacing. In contrast, our hydrogel metapad can achieve a continuous gradient acoustic refractive index through material-level innovation that controls microscopic porosity. This mechanism eliminates the geometric constraints of spatial aliasing, enabling broadband focusing capabilities. Furthermore, the metapad can achieve a significant contrast improvement near the focal region without compromising spatial resolution. Therefore, the metapad holds great potential as a versatile, non-invasive add-on interface for diverse biomedical imaging scenarios.

While acoustic metamaterials offer sophisticated wave control, achieving both transparency and broadband functionality, particularly in aqueous or biological media, remains challenging. These limitations arise primarily from impedance mismatch, absorption, and the inherently narrow bandwidth often associated with resonance-based or macroscopic structural designs. Our hydrogel metamaterials design approach circumvents these issues. It exhibits a constant gradient refractive index over a broad frequency range, enabled by the pore size on the order of microns, making it easy to reach the low-frequency limit required by the multiple-scattering model [49]. Within the investigated frequency range (up to 12 MHz, corresponding to *λ* ≈ 124 μm in water), this condition is well maintained, ensuring a stable effective refractive index. This strategy represents a paradigm shift from manipulating sound via discrete macroscopic inclusions to controlling acoustic properties at a much finer microstructural level. The lower operational frequency bound (0.2 MHz) is instead constrained by the finite aperture of the device. According to classical diffraction theory, effective focusing requires the aperture size (*D*) to be sufficiently larger than the wavelength (*λ*). At 0.2 MHz, the wavelength in water (*λ* ≈ 7.415 mm) results in an aperture-to-wavelength ratio of ∼3.77. This proximity to the critical threshold marks the onset of the diffraction limit. Below this frequency, diffraction effects dominate over refraction, making effective focusing impossible. The microstructurally driven soft design significantly enhances the broadband capabilities of acoustic devices, positioning the metapad as a promising candidate for next-generation ultrasound technologies, thereby facilitating the acquisition of clearer critical medium information.

The broadband focusing performance of the metapad relies on plane wave techniques when used with ultrasound arrays. In the context of ultrafast imaging technology [61–63], which requires the emission of plane waves for real-time imaging, our metapad could direct more acoustic energy to the area of interest, thereby enhancing the contrast. The current metapad focuses plane waves directly within the azimuthal plane, rather than the elevation plane. In ultrasound reception, beamforming is a critical process. Although the metapad introduces inhomogeneities that could render conventional ultrasound reception algorithms, such as the standard delay-and-sum (DAS) approach [61], ineffective in current imaging, the local sound speeds of the designed metapad regions are known. This makes it relatively straightforward to correct for the inhomogeneities and achieve clear, undistorted ultrasound images. More in-depth algorithms adapted to metapad and 3D imaging simulations, considering the elevation plane, will be further investigated in future work. Additionally, although the ray-tracing simulation presented in Fig. S13 serves as a first-order approximation, neglecting media heterogeneity and scattering, the imaging results confirm the metapad’s robustness (Fig. 6). This implies that the gradient index profile exerts a dominant wavefront modulation that effectively overcomes tissue-induced perturbations. Future work may combine full-wave simulations with aberration correction algorithms to further address residual phase distortions [64–66]. Furthermore, while geometric aspherical lenses, horns, and transformation-acoustics designs enable beam control, their rigid, non-conformable shapes or anisotropic structures with limited bandwidth restrict their use in soft-tissue or conformal ultrasound applications. By contrast, embedding the focusing function directly within a soft, biocompatible hydrogel using the gradient-index (GRIN) principle allows the metapad to support compact, flexible, and conformable ultrasound probes and devices. To facilitate the customization of the metapad for various requirements, an inverse design approach relating the structural porosity to the target acoustic field is provided in Supplementary Discussion 11.

The implications of this technology extend beyond imaging of tissues and organs using ultrasound at frequencies above 1 MHz, as the metapad’s excellent ultrasound transparency and broadband focusing performance enable its utilization in acoustic mechanobiology [67–69] and *in vitro* or *in vivo* acoustic manipulation [70–72]. Ultrasound frequencies below 1 MHz are significant in rehabilitation medicine, with typical applications including transdermal drug transport and gene delivery [73,74], adjunctive therapy for wounds [75,76], and ultrasonic neural modulation [77–79]. In these processes, focused ultrasound can deliver energy more efficiently [80], whereas non-focused ultrasound requires higher frequencies and longer durations to achieve the desired effects [81,82]. Thus, due to its broadband characteristics, our metapad holds great potential to enhance ultrasound diagnosis and treatment in clinical medicine. Yet it should be noted that the current study involves a limited sample size and does not cover diverse patient populations (e.g. varying body mass index (BMI)), organs such as the liver, or the human fetus. Future work will focus on extensive clinical validation involving larger cohorts to evaluate the metapad’s performance across different anatomical regions and body types. Furthermore, a detailed analysis of the clinical translation pathway, including regulatory considerations (e.g. National Medical Products Administration (NMPA) registration) and safety feasibility, is provided in Supplementary Discussion 12.

While the metapad’s ability to achieve ultrasonic focusing is undoubtedly appealing, hydrogel metamaterials have the potential to expand the frontiers of wave physics. Whereas most recently reported acoustic metamaterials are designed for a single frequency in a specific application, our hydrogel metamaterials can have various complex broadband functions, including beam steering [53,83,84], vortex beam generation [41,85,86], anti-aberration [87], etc., achieved by manipulating the spatial distribution of polymer networks and pores. Moreover, the design of underwater acoustic metamaterials with acoustic impedance matched to water and low energy loss poses a significant challenge. Simulations conducted within the underwater acoustic frequency range further support the potential of the metapad technology for applications in underwater scenarios (Fig. S17h). The transparent metapad holds the potential to offer novel insights to improve current imaging sonar and echo detection [29]. It should be noted that the present work focuses on wavefront control and does not yet address broadband impedance matching between piezoelectric transducers and water. Future developments could extend the metapad concept by introducing impedance gradients along the acoustic propagation direction, thereby enabling simultaneous broadband impedance matching and wavefront engineering within a unified, soft platform.

The hydrogel metapad may open new avenues in developing next-generation soft acoustic functional devices that can interface with tissue and water environments. Our soft matter techniques pave the way toward the production of soft, programmable, and transparent ultrasound devices. The precisely engineered gradient index metapad also provides a unique testbed for investigating fundamental acoustic wave phenomena, such as refraction in complex media, broadband focusing effects, and wave propagation through anisotropic or gradient soft materials, as required for key topics in acoustic physics.

## METHODS

Detailed materials and methods are available in the online Supplementary data.

### Ethical statements

The studies involving human participants were reviewed and approved by the Ethical Committee of the Clinical Research Ethics Committee of the First Affiliated Hospital of Xiamen University. Written informed consent for participation was not required for this study in accordance with national legislation and institutional requirements. All animal protocols were approved by the Institutional Animal Care and Use Committee of Xiamen University (Approval No. XMULAC20250099).

## Supplementary Material

nwag048_Supplemental_File
