# Supplementary material for: Hydrogel metapad with ultrasound transparency and broadband focusing for biomedical imaging
Source: Natl Sci Rev. 2026 Jan 28;13(6):nwag048. doi: 10.1093/nsr/nwag048 (PMC13007887; doi:10.1093/nsr/nwag048)
Supplement: nwag048_Supplemental_File [file nwag048_supplemental_file.pdf]

Supplementary Data for

Hydrogel metapad with ultrasound transparency and broadband focusing for biomedical imaging

Jinhu Zhang, Deshuai Yu, Tianye Zhang, Chengtian Gao, Erqian Dong, Zhongchang Song, Chaoyu Fan, Zhehao Han, Fabrice Lemoult, Mathias Fink, Zhanxiang Wang*, Youhui Lin*, Yu Zhang*

Corresponding authors. E-mails: yuzhang2025@sjtu.edu.cn; linyouhui@xmu.edu.cn; wangzx@xmu.edu.cn

This PDF file includes:

Experimental Section

Supplementary Discussion

1. Composition of PVA/PAAm DN hydrogel metamaterials
2. Comparison of ultrasound transparency of hydrogel metamaterials, metal-doped polymers and porous silicone rubber
3. Mechanical flexibility and friendliness of the designed hydrogel metamaterials
4. Radial acoustic refractive index distribution of the hydrogel metapad for broadband focusing
5. Theory of sound ray trajectory in the hydrogel metapad
6. Ultrasound focusing capability of the hydrogel metapad
7. Broadband focusing capabilities of metapad in comparison to conventional array techniques
8. Comparison of the metapad and commercial liquid couplant for imaging axial resolution
9. Quantitative effective imaging area for metapad-enhanced ultrasound imaging system
10. Quantitative evaluation of imaging performance
11. An inverse design approach for metapad
12. Regulatory pathway and clinical translation feasibility toward clinical commercialization

Figure S1. Intrinsic limitations on the acoustic transmission capacity and stability of ultrasonic couplants.

Figure S2. Chemical changes in PVA/PAAm hydrogel metamaterials during drying and re-swelling.

Figure S3. Changes in transparency of PVA/PAAm hydrogel metamaterials during drying and re-swelling.
Figure S4. Experimental measurement of acoustic properties.
Figure S5. Experimental verification of the reversible porosity-refractive index relationship across multiple drying-rehydration cycles for the PVA/PAAm double-network hydrogels.
Figure S6. Effects of crystallinity and crosslink density on the acoustic refractive index of hydrogels.
Figure S7. Statistical curve fitting of acoustic attenuation versus porosity for PVA/PAAm hydrogels.
Figure S8. Evaluation of acoustic properties of hydrogel metamaterials and epoxy resin composite metamaterials.
Figure S9. Evaluation of acoustic transmission properties of the metapad and porous silicone lens.
Figure S10. Compressive stress−strain curves of the pure PVA and pure PAAm hydrogels used in the metapad.
Figure S11. Mechanical performance of PVA/PAAm hydrogels constructing the metapad.
Figure S12. Mechanical durability characterization of the metapad constituent hydrogels under cyclic compression.
Figure S13. Acoustic refractive index profile of the hydrogel metapad.
Figure S14. Ultrasound focusing capability of the metapad.
Figure S15. Fabrication and integration of the hydrogel metapad.
Figure S16. Ultrasound focusing capability of the hydrogel metapad through locally high curvature tissue surface.
Figure S17. Broadband characteristics of the hydrogel metapad for ultrasound focusing in penetrating tissues.
Figure S18. Simulations of ultrasound imaging via hydrogel metapad.
Figure S19. Broadband quantitative assessment of imaging resolution and contrast.
Figure S20. Ultrasonographic images and quantitative evaluation of the common carotid artery.
Figure S21. Quantitative Effective Imaging Area (EIA) for metapad-enhanced ultrasound imaging system.
Figure S22. Blood flow in the carotid artery is imaged using (a) the metapad, (b) coupling gel, (c) liquid couplant, and (d) silicone elastomer.
Figure S23. Quantitative evaluation of imaging performance.
Figure S24. Cytotoxicity evaluation of the hydrogel metapad.
Figure S25. Blood and skin contact safety evaluation of the hydrogel metapad.

Table S1. Comparison of physical characteristics of the hydrogel metapad, ultrasound coupling devices, and acoustic functional device.
Table S2. Comparison of acoustic properties of the metapad based on hydrogel metamaterials and common coupling media.
Table S3. Comparison of the hydrogel metapad and existing acoustic focusing lens.
Table S4. Statistical Validation of Theoretical Predictions and Empirical Fits for Acoustic Properties.
Table S5. Acoustic impedance and attenuation coefficients at 0.5, 1, and 2 MHz of the four hydrogels used in the metapad design.
Table S6. Parameters of ultrasound transducer combined with metapad for ultrasound imaging.
Table S7. Acoustic parameters of the discretized metapad for ultrasound imaging.
Table S8. Quantitative comparison of technical specifications between the hydrogel metapad and commercial and flexible Arrays.
Table S9. Parameters used for ultrasound imaging simulation.

Supplementary References

Experimental Section

Materials

Acrylamide and poly (vinyl alcohol) were purchased from Aladdin. Ammonium persulfate and N,N′-methylenebisacrylamide were obtained from Macklin. MEM-α, Cell Counting Kit-8 (CCK-8), Calcein-AM staining kit, and fetal bovine serum (FBS) were purchased from Beyotime (Shanghai, China). MC3T3-E1 cells were purchased from Pricella (Wuhan, China). All chemicals were used directly without purification. Ultrapure water (18.2 MΩ·cm; Millipore Co., U.S.) was used throughout the experiment.

Gradient porosity fabrication of PVA hydrogel metamaterials

PVA solutions were prepared by dissolving PVA powder in deionized (DI) water under vigorous stirring and heating at 100°C for 5 h. To fabricate the three-cycle freeze-thawed PVA (FT PVA) hydrogels with gradient porosity, PVA solutions with different concentrations (5 wt%–15 wt%) were vacuum degassed (at −0.1 MPa) and transferred into a Teflon mold with an acrylic plate cover, and then frozen at −20°C for 8 hours, followed by thawing at room temperature for another 3 hours. The freeze-thawing cycle was repeated twice.

Gradient porosity fabrication of PAAm hydrogel metamaterials

Acrylamide (≥ 99.0%, AAm) as monomer (20 wt%–45 wt%), Ammonium persulfate (APS) as the initiator (0.4 wt% of AAm), and N,N'-methylenebisacrylamide (≥ 99.0%, MBAA) as crosslinker (0.04 wt% of AAm) were dissolved in deionized water to prepare the precursor solution. After being completely vacuum degassed (at −0.1 MPa), clear solutions were obtained and transferred into a Teflon mold with an acrylic plate cover. Thereafter, the PAAm hydrogels were obtained by irradiating UV light (wavelength 365 nm, 60 $mW \cdot cm^{-2}$) for 20 minutes.

Gradient porosity fabrication of PVA/PAAm hydrogel metamaterials

The PVA/PAAm hydrogel was synthesized via a two-stage process. Firstly, the monomer AAm was dissolved in PVA solution (10 wt%), and the mass ratio of AAm to PVA in the solution was varied to construct gradient porosity. In this article, PxAy refers to the mass ratio of PVA to AAm as $x : y$. Then, initiator APS (0.4 wt% of AAm), and crosslinker MBAA (0.04 wt% of AAm) were added to the solution. The mixture was stirred for 0.5 h to ensure homogeneity. The prepared pre-gel solution was vacuum degassed (at −0.1 MPa) and injected into Teflon mold, and covered by an acrylic plate. Polymerization was carried out by irradiating UV light (wavelength 365 nm, 20 W) for 0.3 h. The obtained gel at this stage is denoted as PVA/PAAm stage I. PVA/PAAm stage II was prepared by drying PVA/PAAm stage I overnight at 60°C, and subsequently immersed in DI water at room temperature for several hours as determined by the need for acoustic refractive index.

Measurement of porosity

The porosity was controlled by adjusting the volume of the dispersed water phase. For pure PVA, pure PAAm, and PVA/PAAm Stage I hydrogels, the porosity was defined by the weight of water (m_{w}) used in the preparation of the monomer pre-solution and the total hydrogel volume (V) of the hydrogel. The porosity (φ) was identified as

$$\varphi = \left(m_{\mathrm{w}} / \rho_{\mathrm{w}} \right) / V \times 100\% \tag{S1}$$

The polymerization is conducted in sealed molds to strictly prevent water evaporation, thereby locking in the designed porosity. For the PVA/PAAm stage II hydrogels involving drying and re-swelling, the final porosity is controlled by monitoring the mass of the re-swelled hydrogel until it reaches the target water content.

Measurement of acoustic parameters

The characterization of acoustic parameters, including longitudinal sound speed and attenuation coefficient, was performed strictly in accordance with the national standard GB/T 18022-2000 (*Methods for measuring the longitudinal sound speed and attenuation coefficient of rubbers and plastics*). We employed the insertion substitution method, where the sample was placed in the path of a plane wave sound beam between a transmitting and a receiving transducer in a water tank, replacing an equivalent length of water. The sound speed and attenuation coefficient were derived from the changes in the propagation time and amplitude of the acoustic pulse signal recorded before and after the sample insertion. Fig. S4 shows the experimental setup used for the measurement and data collection of acoustic parameters. Samples of cylindrical hydrogel metamaterial with a radius of 40 mm were placed between two ultrasonic transducers. It should be ensured that the sample surface is perpendicular to the ultrasonic transducer acoustic excitation direction. The system generated pulsed signals of 0.5 MHz, 1 MHz, and 2 MHz through three ultrasonic transducers (HPCTN) positioned below the water surface. The water tank dimensions were designed to be sufficiently large to eliminate interference from reflected waves off the tank walls, ensuring the isolation of the direct pulse signal. A signal generator (AFG 31000 SERIES; Tektronix) was used to excite the transducer. Ultrasound waves propagated through the water path toward the sample. The received signal data were then A/D converted using a digital oscilloscope (MDO32; Tektronix) with a sampling rate of 100 MHz and post-processed by MATLAB scripts. According to the reflection and transmission laws of ultrasonic waves at the interface of two phases and the attenuation law of acoustic pressure, we obtain sound speed (c_{L}) and acoustic attenuation coefficient (α) in hydrogel metamaterial, as shown in the following equation [1]:

$$c_{\mathrm{L}} = \frac{d c_{\mathrm{w}}}{d - c_{\mathrm{w}} \Delta t} \tag{S2}$$

$$\alpha = \frac{1}{d} \left[20 \lg \frac{A_{\mathrm{w}}}{A} - 20 \lg \frac{\left(Z + Z_{\mathrm{w}} \right)^2}{4 Z Z_{\mathrm{w}}} \right] + \alpha_{\mathrm{w}} \tag{S3}$$

where d is the thickness of the sample, $\triangle t$ is the time difference between the received pulse waveform with and without the sample, c_{w} is the speed of sound in water (1483 m/s), A_{w} and A are

acoustic signal amplitudes received by the receiving transducer without and with the sample inserted respectively, α_w is acoustic attenuation coefficient of water at a certain temperature and testing frequency. In this work, the attenuation coefficients of 0.5 MHz, 1 MHz and 2 MHz ultrasound in water were 9.73×10^{-4} dB·cm^{-1}, 4.26×10^{-3} dB·cm^{-1} and 1.54×10^{-2} dB·cm^{-1}, respectively [2]. The sound speed is then multiplied by the density of the sample to obtain the acoustic impedance ($Z = \rho c$). The density (ρ) is obtained by dividing the mass by the volume. The volume of the sample is easily obtained by multiplying the bottom area by the height, since the geometry is standard cylindrical. By measuring multiple samples with the same preparation step, a relatively accurate measurement of the acoustic parameters of samples can be obtained. The acoustic refractive index can be calculated using $n = c_0 / c_{\mathrm{L}}$ where c_0 is the sound speed in the ambient medium (Water, $c_0 = 1483$ m/s at 20°C), and c_{L} is the sound speed of hydrogel metamaterials. During the test, the water temperature was maintained at 20°C.

Mechanical test

The mechanical properties of the designed hydrogel metamaterials were tested using cube (1 cm × 1 cm × 1 cm) specimens. Compression tests were performed at a rate of 10 mm/min using an Instron 5948 mechanical tester. The values of three tests for each sample were averaged to improve measurement precision.

Cell experiments

The MC3T3-E1 cells were cultured in MEM-α containing 10% fetal bovine serum (FBS) in a humidified atmosphere with 5% CO2 at 37°C for 24 hours. The cytotoxicity of four kinds of hydrogels against MC3T3-E1 cells was evaluated using the CCK-8 assay. The extracts of hydrogels were obtained by immersing 50 mg hydrogel in 1 mL FBS-free medium at 37°C for 24 h, and were supplemented with 10% (v/v) FBS for cell culture. MC3T3-E1 cells were seeded into 96-well plates for 24 h. Then, these cells were cocultured with different extracts. After incubation for another 24 h and 72 h, the cytotoxicity was measured using the CCK-8 solution according to the manufacturer's instructions. The absorbance of the solution at 450 nm was recorded using a microplate reader (TECAN, Switzerland). Live cell assay was applied to further evaluate cell proliferation. At the predetermined time, the cells were stained with Calcein-AM. After incubation at 37°C for 20 min, the cells were washed two times with PBS and imaged via a fluorescence microscope (IX71, Olympus).

Hemolytic experiment

The hemocompatibility of the hydrogels was evaluated by a hemolysis assay according to a previously reported method [3]. Briefly, different hydrogel samples were incubated with a 2% erythrocyte suspension at 37°C for 3 h. After incubation, the mixture was centrifuged, and the supernatant was collected and photographed. Hemolysis was assessed by measuring absorbance at 542 nm using a Varioskan™ LUX Multifunctional Microplate Reader.

Skin contact safety test

BALB/c mice were mildly anesthetized using isoflurane. The hair on the dorsal area was carefully removed using an electric shaver. Pre-formed samples (1 cm in diameter) were applied closely to the prepared skin sites and left in place for 6 hours under standard housing conditions. Photographic documentation of the application sites was performed under consistent distance and lighting conditions at three time points: before application (0 hour), during application, and immediately after patch removal at 6 hours. The primary observation endpoints were the presence or absence of irritant reactions such as erythema and edema.

Other general characterization of hydrogel metamaterials

The morphology of freeze-dried hydrogel metamaterial was observed by SEM (FEI Quanta 650FEG). The pore size of various hydrogels was analyzed from SEM images by using ImageJ software. X-ray diffraction (XRD, Bruker D8 ADVANCE) was carried out on the samples in reflection mode (2θ = 15–60°) with a fixed voltage and current (40 kV and 30 mA). Fourier transform infrared spectroscopy (FTIR, PerkinElmer) was carried out using the KBr disc method to obtain spectra in the range of 500–4000 cm^{-1} with 32 scans. The crystallinities of PVA/PAAm were quantified by DSC (Polyma DSC-214). The transmittance study of PVA/PAAm hydrogel metamaterials was characterized by UV-vis spectroscopy (Macylab UV-1800A).

Fabrication and integration of the metapad

To fabricate four discrete layers of hydrogel metamaterials with different acoustic refractive index, four sets of molds were designed consisting of a core layer with a circular ring and three cladding layers with an inner ring (white) and an outer ring (gray) (Fig. S15). SolidWorks 2020 (Dassault Systemes S.A, USA) was used to design models for fabricating metapad, and converted into the format of STL. IdeaMaker (Raise 3D) was used for 3D slicing the model into the format of G-code, which was then imported into a fused deposition modeling (FDM, Raise3D's Pro2 Plus) 3D printer. The PLA filament with a diameter of 1.75 mm is extruded through a 0.4 mm diameter nozzle for the fabrication of 3D printed metapad molds. With these parameters, the fabrication tolerance was strictly controlled within ±0.2 mm. The core layer was obtained by injecting 10 wt% PVA solution into the first set of molds after 3 cycles of freeze-thawing. The 30 wt% AAm solution mixed with initiator and crosslinker was injected into the second set of molds and polymerized by UV light to obtain cladding 1. The third and fourth sets of molds were injected with P1A6 solution. Cladding 2 was directly polymerized by UV irradiation. To ensure geometric consistency, all the polymerization processes are conducted in molds sealed with an acrylic plate to strictly ensure dimensional accuracy during crosslinking. For cladding 3, we first fully dried P1A6 stage I in the fourth set of molds and transferred it to the fifth set of molds to obtain P1A6 stage II after re-swelling. The stage II hydrogel was then manually trimmed guided by a rigid circular template to the designed dimensions. Although this manual process introduces slight variations compared to mold-casting, repeated measurements confirmed that the dimensional tolerance was controlled

within ±0.5 mm. Finally, in order to avoid strong acoustic scattering by air trapped in the interface, four layers of hydrogel metamaterials were integrated underwater to obtain the metapad.

The fabricated metapad has a total thickness of 1 cm. The radial structure is divided into distinct concentric regions: a core layer (radius r = 0–5.3 mm) with a refractive index of n = 1.026; cladding layer 1 (r = 5.3–9 mm) with n = 1.113; cladding layer 2 (r = 9–11.4 mm) with n = 1.200; and cladding layer 3 (r = 11.4–17 mm) with n = 1.348.

Finite element simulations of acoustic field with the metapad

The 3D finite element model solved the homogeneous Helmholtz equation for the scattered pressure field from an incident plane wave defined in the computational domain (COMSOL Multiphysics, Stockholm, Sweden). In the fluid media, only longitudinal waves will propagate, which can be described by the homogeneous Helmholtz equation [4]

$$\nabla \cdot \left(-\frac{1}{\rho} \nabla p \right) - \frac{1}{\rho_0} \left(\frac{w}{c} \right)^2 p = 0 \tag{S4}$$

where p is the sound pressure, ρ_0 is the density, and c is the sound velocity. The acoustic fields were numerically derived by solving the above equations. Both hydrogels and porcine skin were modeled approximately as incompressible linear elastic materials with Poisson's ratio v = 0.5 in which only longitudinal waves propagate. The surrounding fluid in this model was water. In addition, the Perfectly Matched Layer (PML) was set in the outermost layer of the computational domain, which is an artificial non-reflecting and absorption layer absorbing all outgoing waves that mimics a domain stretching to infinity.

Focusing simulation parameters for conventional array

The array transducer used in this simulation consists of 96 elements, each with a width of 0.483 mm and an inter-element kerf spacing of 0.03 mm, resulting in a total element pitch of 0.513 mm. The overall aperture size of the probe is 50 mm. The designed focal length was fixed at 40 mm.

Ultrasound focusing experiments of the metapad

Fig. 5a shows the experimental setup for ultrasound transmission through porcine skin via metapad modulation. The metapad and ultrasound probe (HPCTN-1-20-I-B, with a diameter of 28 mm) were fixed together with a 3D printed holder. Ultrasound transmission experiments were performed in a water tank with dimensions of 2 m × 1.2 m × 0.8 m. The 3D moving motor, with a minimum accuracy of 1 μm, has been used for sound field scanning experiments. A five-cycle sinusoidal burst of 1 MHz was generated using a waveform generator (AFG 31000 SERIES; Tektronix), and the signal was amplified by a power amplifier (ATA-4011, Aigtek). It is noted that the measured acoustic waveform exhibits a duration exceeding the five-cycle electrical excitation. This elongation is attributed to the transducer's inherent electromechanical ring-down effect. The signal power was kept consistent in the comparison experiments. We then evaluate the ultrasound focusing performance using fresh porcine skin as the model tissue. The metapad itself functions as a coupling medium. The metapad was in direct contact with the probe and the porcine skin. The

lower surface of the porcine skin was submerged in water. The porcine skin was held in place with 3D-printed brackets and screws. The needle hydrophone (ZS-1000, Maihuang Technology) with a sensitivity of 0.099 μV/Pa at 1 MHz was used to record the ultrasound signal in the scanning area. According to the calibration certificate, the expanded measurement uncertainty (U) of the sensitivity calibration with a coverage factor k = 2 (95% confidence level) is 2.0 dB in the range of 400–1000 kHz and 2.5 dB in the range of 2–5 MHz. This corresponds to a pressure amplitude uncertainty of approximately ±26% to ±33%. Synchronization was achieved by connecting the TTL sync output of the waveform generator directly to the external trigger input of the digital oscilloscope. This ensured precise time-alignment for the point-by-point scanning. The signal was acquired by a digital oscilloscope and transferred to the computer by USB interface. During this test, the water temperature was at room temperature (about 20–25°C).

Ultrasound imaging simulation

The simulations of plane-wave imaging were performed using the Field II simulation program. We modeled a linear array transducer consisting of 192 elements with a center frequency of 7.5 MHz and an element pitch of 0.2 mm. The transducer excitation consisted of a one-period square wave, and the impulse response of both the emission and reception apertures was a two-cycle Gaussian-modulated sinusoidal pulse. Transmit and receive apodization were set to Hanning windows, and the sampling frequency was 100 MHz to ensure calculation accuracy. Beamforming employed a lateral scan spacing of $\lambda/2$, corresponding to 0.1 mm, with a total of 384 lines. The detailed parameters used for ultrasound imaging simulation are listed in Table S9. To model the focusing effect of the metapad within the Field II environment, the phase delay profile derived from the gradient refractive index distribution was calculated and superimposed onto the transducer's transmit delay laws. Regarding image reconstruction, we implemented a modified Delay-and-Sum (DAS) algorithm for receive beamformer. Unlike standard DAS which assumes a uniform speed of sound (c_0), our approach incorporated an additional phase delay compensation term ($\triangle\tau$). These delays were calculated based on the spatial refractive index profile of the metapad to compensate for the time-of-flight variations as acoustic waves traversed the gradient index layer. This correction effectively compensated for the wavefront distortion induced by the metapad, resulting in artifact-free images with accurate target localization. Attenuation was applied to the phantom, using a typical soft tissue value of 0.5 dB/cm/MHz [5]. Two artificial phantoms were used: one consisting of a collection of point targets and the other comprising 12 anechoic cyst regions. These phantoms serve to characterize the resolution and contrast-lesion detection capabilities of the imaging system with our metapad. Homogeneous tissue was mimicked using a collection of randomly placed scatterers with Gaussian-distributed scattering strengths.

The lateral resolution was determined by the point-spread function (PSF). An ultrasonic image is typically represented as being generated through the PSF function, linking the object to the image $o(x_1, z_1)$ with the obtained image $s(x, z)$ [6]:

$$s(x,z) = \iint h(x,z,x_1,z_1)\, o(x_1,z_1)\, \mathrm{d}x_1 \mathrm{d}z_1 \tag{S5}$$

The PSF function can be obtained considering that $h(x, z, x_1, z_1)$ is the image of a point object placed in (x_1, z_1). We used Field II to make this calculation for a number of points simultaneously, and thereby the −10 dB width of the PSF directly reflects the lateral resolution.

The contrast was computed as follows [7]:

$$Contrast = 10\log_{10}\frac{\int_{x,z\in D_{\mathrm{out}}}\left|h(x,z)\right|^2\mathrm{d}x\mathrm{d}z}{\int_{x,z\in D_{\mathrm{in}}}\left|h(x,z)\right|^2\mathrm{d}x\mathrm{d}z} \tag{S6}$$

The numerator represents the background signal and is determined as the mean pixel value within the region of interest (ROI) near the inclusion (D_{out}, 10 × 10 pixels). The denominator, representing the anechoic inclusion signal, is calculated as the mean pixel value within the ROI inside the inclusion area (D_{in}, same size as D_{out}).

Ultrasound imaging of human organs

The current study involved a single healthy volunteer (a 25-year-old male) with multiple independent acquisitions to demonstrate technical reproducibility. All human experiments were conducted under approved ethical protocols; detailed approval information is provided in the Acknowledgments section. To ensure clinical safety, real-time indices were monitored. Recognizing that commercial systems calculate indices based solely on transducer output, we deliberately operated at low baseline levels to accommodate the metapad's passive gain. Specifically, carotid (MI=0.33, TIS=0.60) and cardiac (MI=0.78, TIS=0.30) imaging were conducted at settings providing ample safety margins. This ensured that the effective in situ acoustic pressure remained significantly below the FDA diagnostic threshold (MI < 1.9), precluding cavitation or thermal risks.

Supplementary Discussion

1. Composition of PVA/PAAm DN hydrogel metamaterials

As shown in Fig. S2a, there are abundant amide groups on the PAAm chain, which can form hydrogen bonds with the hydroxyl groups on the PVA chain for tightly crosslinking. The Fourier transforms infrared (FTIR) spectrum of the PVA/PAAm DN hydrogel are exhibited in Fig. S2b. The characteristic peaks at 3324 cm^{-1} and 3194 cm^{-1} correspond to the -OH and -NH groups from the PVA/PAAm DN hydrogel, respectively. The peak at 1654 cm^{-1} represents the C=O stretching, the peak at 1610 cm^{-1} is attributed to the –NH deformation of the amide, and absorption at 1091 demonstrates the crystalline region of PVA. The XRD patterns of PVA, PAAm and PVA/PAAm were shown in Fig. S2c. The profile of PVA/PAAm hydrogel shows a strong diffraction peak at 2θ = 19.5°, which are attributed to the characteristic diffraction reflections of crystalline PVA. In addition, the PAAm hydrogel presents a broad noncrystalline diffraction peak centered at 2θ = 24°, which can also be observed in the profile of PVA/PAAm hydrogel. The above analysis illustrates the composition of PVA/PAAm DN hydrogel metamaterials.

2. Comparison of ultrasound transparency of hydrogel metamaterials and metal-doped polymers

The closer the acoustic impedance matches the environment, the lower the absorption and attenuation, resulting in greater acoustic transparency in that environment. Resin composites, such as tungsten-epoxy composites, are known to adjust the refractive index of sound waves by doping metal powders into the polymer matrix [8]. For a fair comparison, we ensured that the tungsten-epoxy composites, PAAm hydrogel, and PVA/PAAm hydrogel selected for comparison possess the same acoustic refractive index (n = 0.85 relative to water). The density of tungsten-epoxy composite is 2815.3 kg/m^3.

Firstly, a comparison of acoustic transmission loss in water environment was performed. Assuming that a sample with density ρ_2 and sound velocity c_2 is placed in a medium with density ρ_1 and sound velocity c_1, the transmission coefficient (T) and transmission loss (TL) can be determined as [4]

$$T = \frac{4}{4\cos^2 k'_{\text{sample}}d + \left(\frac{Z'_{\text{sample}}}{Z'_{\text{water}}} + \frac{Z'_{\text{water}}}{Z'_{\text{sample}}}\right)^2 \sin^2 k'_{\text{sample}}d} \tag{S7}$$

$$TL = 10\log\left(T\right) \tag{S8}$$

where $Z'_{\text{water}} = \rho_1 c_1 / \cos\left(\theta_{\text{i}}\right)$, $Z'_{\text{sample}} = \rho_2 c_2 / \cos\left(\theta_{2\text{t}}\right)$, $k'_{\text{sample}} = k_{\text{sample}} \cos\left(\theta_{2\text{t}}\right)$, θ_{i} are the incident angles of the sound waves, respectively, θ_{t} are the refraction angles within the sample, and d is the thickness of the sample. The results show that our designed hydrogel metamaterials exhibit extremely low transmission loss (≤ 0.8508 dB) at different incident angles over a wide frequency range (Fig. S8a–c).

Secondly, the attenuation coefficients of the three materials were measured. From the

waveforms, it can be visually observed that after transmitting 500 kHz ultrasound waves through PAAm hydrogel and PVA/PAAm hydrogel, the amplitude of the ultrasound waves shows negligible attenuation (Fig. S8d). In contrast, after transmission through epoxy resin composite materials, the amplitude decreases significantly, demonstrating high absorption attenuation. Further data processing reveals that the hydrogel metamaterial has an acoustic attenuation coefficient that is two orders of magnitude lower than that of the epoxy resin composite material (Fig. S8e).

Then, we also evaluated acoustic properties of the metapad and porous silicone lens. Compared to the porous silicone lens with an attenuation coefficient of 34.45–96.20 dB/cm at 0.2 MHz, the metapad achieves much lower attenuation, just 0.02–0.38 dB/cm at 1 MHz (Fig. 3e). Additionally, the metapad offers superior impedance matching with water or tissue, ensuring minimal acoustic transmission loss across a broad frequency range (Fig. S9).

3. Mechanical flexibility and friendliness of the designed hydrogel metamaterials

Fig. S11b and c present the compressive stress-strain curves of PVA/PAAm of stage I and stage II hydrogels and the elastic modulus calculated from the curves, respectively (Fig. S11d and e). In stage I, the dissolved PVA was simply mixed with the cross-linked PAAm hydrogel, which did not contribute to the compressive stress, exhibiting a low elastic modulus and a gradual increase in modulus with increasing acrylamide content. However, the stress-strain curves of the hydrogels obtained in stage II exhibit the reverse trend after drying and re-swelling. The increase in the ratio of PVA to acrylamide resulted in a gradual increase in modulus, which is attributed to the crystallization of PVA. From the macroscopic images, transparency of PVA/PAAm hydrogels changed obviously from transparent (stage I) to opaque white (stage II), which was consistent with the result of transmittance test by UV-vis spectrophotometer (Fig. S3). The crystallization of PVA was further confirmed with DSC as shown in Fig. S2d, and the distinct endotherm melting peaks could be observed at 245°C for PVA/PAAm hydrogels. It leads to the formation of another physically crosslinked network in the PVA/PAAm hydrogels, which greatly improves the mechanical properties of the hydrogels. The elastic modulus of pure PVA and PAAm hydrogels is also dependent on the polymer concentration.

4. Radial acoustic refractive index distribution of the hydrogel metapad for broadband focusing

The focused sound quality through conventional Fresnel lens is usually limited by the spherical/cylindrical aberration. The aberration will focus the incident sound rays at many different points, resulting in a blurred image, whereas with a low-aberration lens, all the different sound rays will focus on a fixed point, yielding a less blurred and higher quality image in comparison. This helps to improve the resolution of ultrasound imaging and increases the focusing energy in HIFU. In this article, a modified hyperbolic secant index profile (red curve in Fig. S13a) is used for the metapad with gradient refractive index design to focus ultrasound without aberration. As shown in Fig. 4b, the hydrogel metapad consisting of a core layer and three cladding layers

assembled by hydrogel metamaterials possesses a radial acoustic refractive index distribution following

$$n(r) = n_0 \operatorname{sech}\left[g(\alpha r)\right] \tag{S9}$$

where $g(\xi) = \xi \big/ \left(1 + \beta_1 \xi^2 + \beta_2 \xi^4\right)$. The outline of the metapad is shown in Fig. S13b. It has a thickness (d) of 1 cm. Although hyperbolic secant profile (black curve in Fig. S13a) has been widely used to design the focusing lens, it still suffers from significant ray aberration that can be observed in the ray trajectories (Fig. S13c). The modified ray trajectories exhibit almost no aberration (Fig. S13d) by introducing correction factors β_1 and β_2. Numerical simulations led to the choice of $\beta_1 = -0.148$ and $\beta_2 = -0.011$.

5. Theory of sound ray trajectory in the hydrogel metapad

The focusing performance of the hydrogel metapad is well described by an analytical model based on ray theory. Ultrasound waves in a non-uniform medium can be described by the wave equation

$$\nabla^2 p(\boldsymbol{r}, w) - \nabla \ln \rho(\boldsymbol{r}) \cdot \nabla p(\boldsymbol{r}, w) + k^2 p(\boldsymbol{r}, w) = 0 \tag{S10}$$

where $k = w / c(\boldsymbol{r})$. Suppose that Equation (S10) is solved as

$$p(\boldsymbol{r}, w) = A(\boldsymbol{r}, w) \exp\left[ik_0 S(\boldsymbol{r}, w)\right] \tag{S11}$$

where $k = w / c_0$. Taking Equation (S11) into Equation (S10) and making a high frequency approximation: $k_0 \to \infty$, we can obtain

$$(\nabla S)^2 = n^2(\boldsymbol{r}) \tag{S12}$$

where ∇S is the normal vector of the isophase plane, and can be written as $\nabla S = n(\boldsymbol{r})\boldsymbol{s} = n(\boldsymbol{r})\frac{\mathrm{d}\boldsymbol{r}}{\mathrm{d}s}$.

Derivation of the above equation yields the Eikonal equation

$$\frac{\mathrm{d}}{\mathrm{d}s}\left[n(\boldsymbol{r})\frac{\mathrm{d}\boldsymbol{r}}{\mathrm{d}s}\right] = \nabla n(\boldsymbol{r}) \tag{S13}$$

Due to the fact that the acoustic refractive index n is a function only of r, the trajectories of a normally incident wave can be derived by solving Equation (S13) (Fig. S13b). Specifically, we can obtain

$$\begin{aligned} \frac{\mathrm{d}}{\mathrm{d}s}\left[n(r)\frac{\mathrm{d}z}{\mathrm{d}s}\right] &= 0 \\ \frac{\mathrm{d}}{\mathrm{d}s}\left[n(r)\frac{\mathrm{d}r}{\mathrm{d}s}\right] &= \frac{\mathrm{d}n(r)}{\mathrm{d}z} \end{aligned} \tag{S14}$$

Due to $\mathrm{d}s = \sqrt{(\mathrm{d}z)^2 + (\mathrm{d}r)^2} = \sqrt{1 + (r')^2}\,\mathrm{d}z$ when only considering the two-dimensional case, the above equation can be derived as:

$$\frac{n(r)}{\sqrt{1+(r')^2}}=n(r_0) \tag{S15}$$

Based on this, the focal length of metapad is

$$focal\ length=y_h\sqrt{\frac{1}{n^2(y_h)-n^2(y_0)}-1} \tag{S16}$$

And then, we consider a hyperbolic secant index profile:

$$n(r)=n_0\,\mathrm{sech}(\alpha r) \tag{S17}$$

where n_0 and α are constants. This profile, also known as a Mikaelian lens [9], is often used to design for low aberration. The ray trajectory is

$$r(z)=\frac{1}{\alpha}\sinh^{-1}\left[\sinh(\alpha r_0)\cos(\alpha z)\right] \tag{S18}$$

For a small αr_0, we can derive a simplified ray trajectory $r(z)=r_0\cos(\alpha z)$. To reduce the aberration, we used a modified hyperbolic secant profile [10] by stretching the y-coordinate in this paper:

$$n(r)=n_0\,\mathrm{sech}\left[g(\alpha r)\right] \tag{S19}$$

where $g(\xi)=\xi\big/\left(1+\beta_1\xi^2+\beta_2\xi^4\right)$. Similarly, for a small $g(\alpha r_0)$, the ray trajectory of our metapad can also be simplified as $r(z)=r_0\cos(\alpha z)$. Therefore, we can further obtain

$$focal\ length\approx\frac{\cot(\alpha h)}{n_0\alpha}+d \tag{S20}$$

As can be seen from the above formula, the focal length of the metapad decreases as its thickness (*h*) increases, which is consistent with our simulation results (Fig. 4f and g).

6. Ultrasound focusing capability of the hydrogel metapad

From the amplitude profiles extracted at *r* = 0 mm and *z* = 39 or 38 mm (Fig. S14a and b), the focusing capability of the metapad was fully characterized, exhibiting full widths at half maximum (FWHM) of 1.217λ_0 and 16.285λ_0 along the *r*-axis (Fig. S14c) and *z*-axis (Fig. S14d), respectively. The experimental results differ slightly from the simulation due to the small Fresnel number of the hydrogel metapad causing the focal shift, discretization of the acoustic refractive index, and non-flat phase on the transducer surface. Despite these influences, the simulation and experiment results are qualitatively consistent.

7. Broadband focusing capabilities of metapad in comparison to conventional array techniques

Array-based techniques widely used in ultrasound imaging are fundamentally constrained by the performance of individual array elements. The wavelength-comparable element size induces spatial aliasing effect due to the violation of the Nyquist criterion, thereby restricting broadband

acoustic wave control [11]. Specially, at higher operating frequencies where the wavelength becomes smaller than the element size and spacing, constructive interference can be achieved at the intended focal point. However, owing to the periodic arrangement of the array and the inherent diffraction characteristics of waves, constructive interference may also occur at other spatial locations. This results in degraded focusing performance, manifesting as focal spot distortion and grating lobes.

A phased array focuses energy at a target point P_{F} by ensuring the coherent superposition of waves emitted from each of its N elements. This is achieved by applying a calculated phase shift ϕ_n to the signal of the *n*-th element. This phase shift precisely compensates for the propagation time from the element to the focal point. Let the distance from the *n*-th element (at position x_n to the focal point P_{F} be $R_{n\mathrm{F}}$. For the waves to arrive at P_{F} in phase, the applied phase shift must be:

$$\phi_n = kR_{n\mathrm{F}} \tag{S21}$$

where $k = 2\pi/\lambda$ is the wavenumber. The total field amplitude at an arbitrary point P is then a superposition of these phase-compensated waves:

$$U(P) = \sum_{n=1}^{N} \frac{A_n}{R_n(P)} e^{j(kR_{n\mathrm{F}} - kR_n(P))} \tag{S22}$$

At the target focus ($P = P_{\mathrm{F}}$), the phase term becomes zero for all elements, resulting in maximum constructive interference. However, there may be other points $P_{\mathrm{G}} \neq P_{\mathrm{F}}$ that can arise a spurious focus or a grating lobe. This happens if the phase difference of the field contributions from adjacent elements is an integer multiple of 2π. The phase difference between element *n*+1 and *n* at a point P_{G} is:

$$\Delta\Psi(P_{\mathrm{G}}) = [kR_{(n+1)\mathrm{F}} - kR_{(n+1)\mathrm{G}}] - [kR_{n\mathrm{F}} - kR_{n\mathrm{G}}] = 2\pi m \tag{S23}$$

where *m* is a non-zero integer (*m* = ±1, ±2, …). This is the fundamental condition for the formation of a spurious focus. The path difference between adjacent elements to a point at an angle *θ* can approximately be $\Delta R \approx d \sin\theta$. Applying this to the spurious focus condition yields:

$$k(d\sin\theta_{\mathrm{F}}) - k(d\sin\theta_{\mathrm{G}}) \approx 2\pi m \tag{S24}$$

Substituting $k = 2\pi/\lambda$ and simplifying, we obtain the equation for the direction of spurious focus:

$$\sin\theta_{\mathrm{G}} \approx \sin\theta_{\mathrm{F}} - m\frac{\lambda}{d} \tag{S25}$$

Therefore, the existence of grating lobes is exclusively determined by the ratio of the wavelength to the element spacing λ/d. If the spacing *d* is large (e.g., $d > \lambda$), it is trivial to find a non-zero integer *m* for which a real angle θ_{G} exists. This leads to the formation of one or more spurious focus spots, diverting energy from the intended target P_{F} and distorting the ideal focus point.

8. Comparison of the metapad and commercial liquid couplant for imaging axial resolution

Ultrasound imaging was performed on the common carotid artery (CCA) of a 25-year-old male subject using the hydrogel metapad as the couplant and we extracted the luminal diameter (LD) and intima-media thickness (IMT) (Fig. S20). Three independent acquisitions were performed to assess measurement repeatability. The results showed that the mean LD with standard deviations was 6.321±0.034 mm (Fig. S20a and b), which is reasonable compared with systematic computed tomography angiographic data (6.5±0.6 mm) [12]. The consistency of the luminal diameter of the CCA in our images aligns with anatomical evidence. Additionally, the mean IMT was 0.586±0.011 mm, which is also reasonable given that the normal value of IMT is about 0.4 mm at birth and 0.8 mm at 80 years in the absence of vascular pathologies [13]. The extremely low standard deviation (± 0.011 mm, corresponding to a coefficient of variation of ~1.9%) underscores the high repeatability of the metapad-enhanced system. The metapad's focusing capability enhances the echogenicity of the arterial boundaries, ensuring that measurements remain consistent across multiple trials.

Furthermore, a sonologist performed the LD and IMT measurements on the same subject using an ultrasound imaging system (LOGIQ E9, GE HealthCare) with a built-in measurement tool (Fig. S20c and d). Commercial liquid couplant (TM-100; Jinya) was used for this measurement. We used this as a standard test, with the results serving as the control group.

9. Quantitative effective imaging area for metapad-enhanced ultrasound imaging system

The metapad functions as a passive gain element that redistributes acoustic energy, creating a spatially heterogeneous intensity map. Conventional definition based on a single lateral width (e.g., −6/−10 dB beamwidth) is insufficient for characterizing the unique performance of the metapad-enhanced system. Standard FOV definitions, which apply well to uniform scanning arrays, are not applicable to the metapad. A simple lateral width metric at the focus underestimates the metapad's utility, as it neglects the broader region where signal intensity remains sufficient for tissue visualization.

To rigorously quantify the usable FOV, we could introduce the concept of "Effective Imaging Area (EIA)". This is defined as the total spatial area within the Region of Interest (ROI) where the signal intensity exceeds a specified dynamic range threshold relative to the peak. Taking Fig. 6d as an example, we statistically analyzed the EIA using four distinct thresholds (Fig. S21). The Effective Imaging Area for signal intensities > −40 dB, > −35 dB, > −30 dB, and > −25 dB was measured to be 332 mm^2, 274 mm^2, 187 mm^2, and 65 mm^2, respectively. This area-based metric demonstrates that while the metapad concentrates energy for high contrast, it maintains a substantial effective coverage for the ROI.

10. Quantitative evaluation of imaging performance

To further evaluate the imaging performance with the metapad, we performed a comprehensive quantitative analysis comparing the proposed metapad (Case 1) against the standard coupling gel

(Case 2) and liquid couplant (Case 3). The evaluation focused on three key performance metrics: axial resolution, acoustic signal intensity, and contrast-to-noise ratio (CNR).

a) Resolution Assessment: We analyzed the signal intensity profile perpendicular to the carotid artery wall (indicated by the yellow line in Fig. S23a). The vessel wall signal exhibits an asymmetric distribution due to the strong echo of the surrounding tissue on one side and the weak echo of the blood flow on the other (Fig. S23c). Therefore, we assessed the resolution by measuring the Half-Width at Half-Maximum (HWHM) on the lumen side (the side transitioning to the blood flow), defined as HWHM = $|X_{\mathrm{half}} - X_{\mathrm{peak}}|$. This edge response effectively reflects the Point Spread Function (PSF) of the imaging system. The measured HWHM values were 0.990 mm for the Metapad (Case 1), 1.047 mm for the coupling gel (Case 2), and 0.914 mm for the liquid couplant (Case 3) (Fig. S23c). These results indicate that the metapad maintains a reliable axial resolution comparable to that of liquid couplants, ensuring high-quality imaging performance in practical applications.

b) Echo Intensity Analysis: To quantitatively evaluate the acoustic energy transmission, we defined a Region of Interest (ROI) at the acoustic focal zone within the carotid artery images (indicated by the white dashed line in Fig. S23a). We used the Mean Gray Value (MGV) of the ROI to evaluate the echo signal intensity. The results demonstrate that the metapad (Case 1) yielded a significantly higher MGV of 143.264 (a.u.). This represents a substantial enhancement compared to both the liquid couplant (Case 3, 103.268) and the coupling gel (Case 2, 66.221) (Fig. S23d). The superior MGV in Case 1 confirms the acoustic focusing capability of the metapad, which effectively maximizes energy transmission to the target tissue.

c) Contrast-to-Noise Ratio (CNR) Analysis: In the heart ultrasound images, we evaluated the image quality by calculating the Contrast-to-Noise Ratio (CNR). The CNR was calculated using the following equation [14]:

$$CNR = \frac{|\mu_{\mathrm{tg}} - \mu_{\mathrm{bg}}|}{\sigma_{\mathrm{bg}}}$$

where μ_{tg} and μ_{bg} represent the mean gray values of the target structure and the background, respectively, and σ_{bg} denotes the standard deviation of the background noise. We defined the Regions of Interest (ROIs) on the target structure and the ventricular cavity (indicated by the yellow squares (10 × 10 pixels) in Fig. S23b). The calculations indicate that the Metapad (Case 1) significantly improved the CNR to 183.22. This represents a substantial enhancement compared to both the coupling gel (Case 2, 31.53) and the liquid couplant (Case 3, 94.394) (Fig. S23e). This result confirms that the metapad provides superior image contrast within the focal region, yielding a clearer distinction against the surrounding tissue.

d) Doppler Velocity SNR Analysis: The Doppler velocity SNR was quantified from the spectral Doppler waveforms (Fig. S22). We measured the mean pixel intensity (gray values) of the spectral envelope at peak systole (A_{signal}) and the background noise floor

(A_{noise}). The SNR was calculated as

$$SNR = 20\log_{10}\left(\frac{A_{signal}}{A_{noise}}\right)$$

The Doppler velocity SNR increased from 24.91 dB (using coupling gel) and 42.04 dB (using liquid couplant) to 80.54 dB with the metapad. This significant SNR improvement could reduce background haze and facilitating cleaner envelope detection.

11. An inverse design approach for metapad

A tailored inverse design approach that correlates structural porosity with specific target acoustic field requirements:

a) Gradient Parameter Determination: The refractive index distribution is governed by the modified hyperbolic secant profile $n(r) = n_0 \,\mathrm{sech}\left[g(\alpha r)\right]$, For a target focal length *F*, the required α can be derived by inverting the ray-acoustic trajectory equation (Equation (S20)): $focal\ length \approx \frac{\cot(\alpha h)}{n_0 \alpha} + d$.

b) Refractive Index Mapping: With the determined α and optimized aberration-correction coefficients (β_1, β_2), the continuous refractive index profile is generated using the modified formula: $n(r) = n_0 \,\mathrm{sech}\left[\frac{\alpha r}{\left(1 + \beta_1 (\alpha r)^2 + \beta_2 (\alpha r)^4\right)}\right]$.

c) Porosity Inversion: Using the Waterman-Truell model, we can map the target $n(r)$ to the required porosity distribution $\varphi(r)$. Since the porosity-index relationship is monotonic, a unique porosity value is assigned to each radial position.

d) Discretization Strategy: The continuous $n(r)$ profile is discretized into concentric rings. We can employ a scheme of equal intervals of sound velocity to obtain a more concentrated focal spot and greater acoustic energy at the focus.

12. Regulatory pathway and clinical translation feasibility toward clinical commercialization

We anticipate following the National Medical Products Administration (NMPA) regulatory pathway in China, classifying the metapad as a Class II medical device. The feasibility of achieving regulatory clearance is supported by the findings of this study:

a) Biocompatibility (GB/T 16886 / ISO 10993): Regarding cytotoxicity, CCK-8 assays on MC3T3-E1 cells demonstrated that cell viability remained above 90% after 3 days of incubation, meeting the fundamental requirements. For hemocompatibility, the hemolysis assay results showed a hemolysis rate of less than 5%, which falls within the safe range specified by international standards. To assess irritation and skin tolerance, we performed a continuous 6-hour contact test on a murine model, where observations

revealed no signs of erythema, edema, or adverse reactions. These quantitative results provide a strong indicator of the material's potential to pass the comprehensive biological evaluation.

b) Performance and Safety Benchmarking: The measured Mechanical Index (MI) at the focal point with the metapad is approximately 0.36 in our focusing experiment, which is significantly lower than the standard safety limit for diagnostic ultrasound (MI < 1.9) specified in GB 9706.237 (equivalent to IEC 60601-2-37). Additionally, the material's extremely low attenuation prevents device self-heating. The study also demonstrated that the metapad can improve imaging contrast and functions effectively as an acoustic coupling layer, providing solid evidence for passing the NMPA performance registration testing.

c) Manufacturing Controls (GMP): Our study verified that a discretized structure achieves focusing performance comparable to an ideal continuous gradient model. This finding indicates that the design is robust to manufacturing steps, facilitating reproducible Quality Control (QC) under Medical Device Good Manufacturing Practice (GMP) standards.

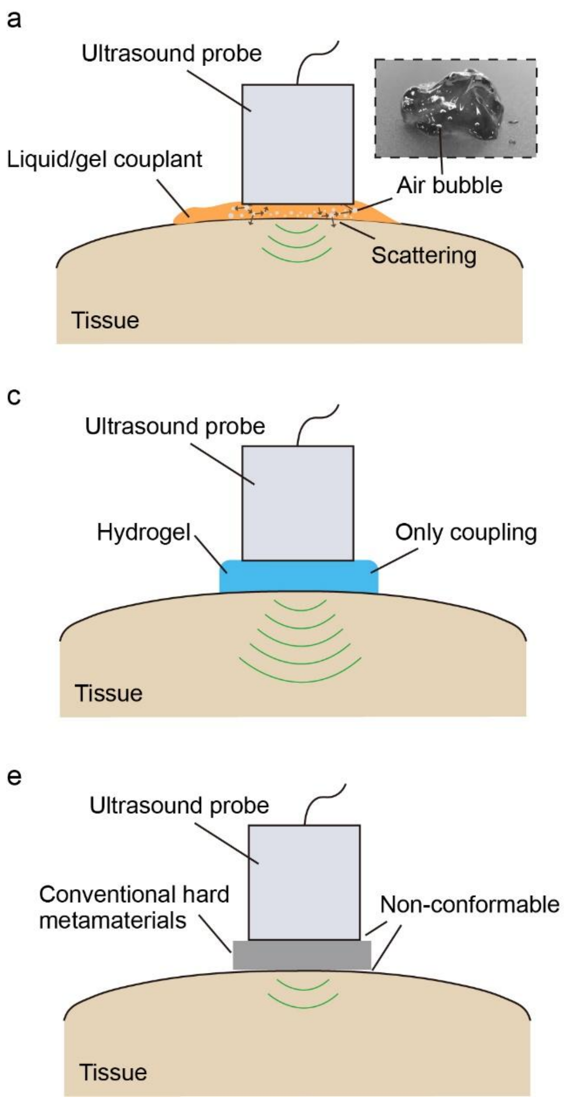

Figure S1. Intrinsic limitations on the acoustic transmission capacity and stability of ultrasonic couplants. (a) The liquid/gel-based couplants of amorphous fluid applied to clinical imaging areas are non-uniform in thickness and can easily trap bubbles, leading to scattering that can form umbrella artifacts. (b) The acoustic impedance of dry couplants (e.g., elastomers including silicone rubber and polystyrene) does not match the tissue resulting in weaker acoustic energy entering the tissue, making it difficult to image deep organs. (c) Conventional hydrogels only have the function of coupling. (d) Thermoplastic polymers, such as polyurethane and epoxy, have a high coefficient of acoustic absorption, resulting in high attenuation of energy. (e) Conventional metamaterials, mostly constructed of hard materials, are not conformal to soft tissues, high curvature areas, and ultrasound probes. (f) The metapad is made of hydrogel metamaterials that uniquely integrates both ultrasound transparency and focusing, which has not been achieved by previous ultrasound devices.

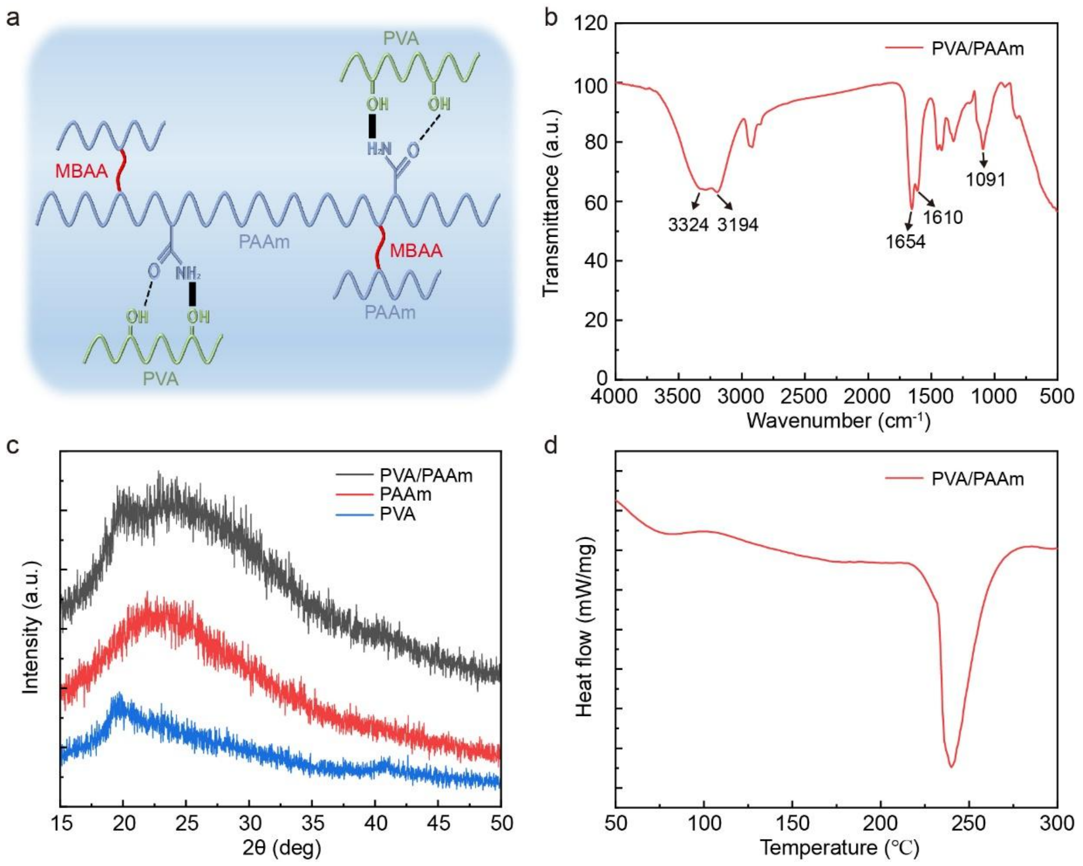

Figure S2. Chemical changes in PVA/PAAm hydrogel metamaterials during drying and re-swelling. (a) Illustration of the network structure of prepared PVA/PAAm DN hydrogels. (b) FTIR spectra of the PVA/PAAm DN hydrogels. (c) XRD patterns of PVA, PAAm and PVA/PAAm hydrogels. (d) Differential scanning calorimetry (DSC) curves of PVA/PAAm.

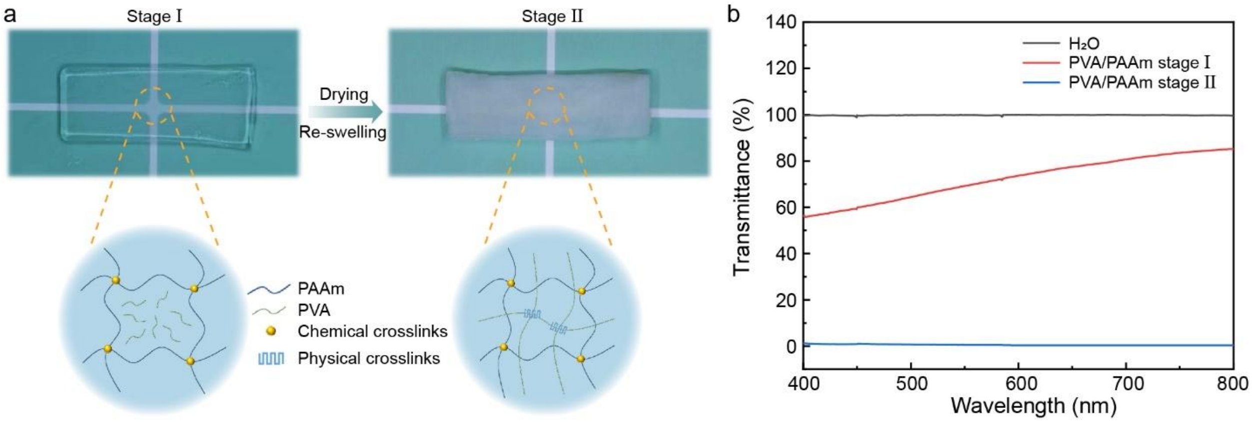

Figure S3. Changes in transparency of PVA/PAAm hydrogel metamaterials during drying and re-swelling. (a) Photograph of PVA/PAAm hydrogel metamaterials in different stage. (b) UV-vis spectrum of the PVA/PAAm hydrogel metamaterials in different stages.

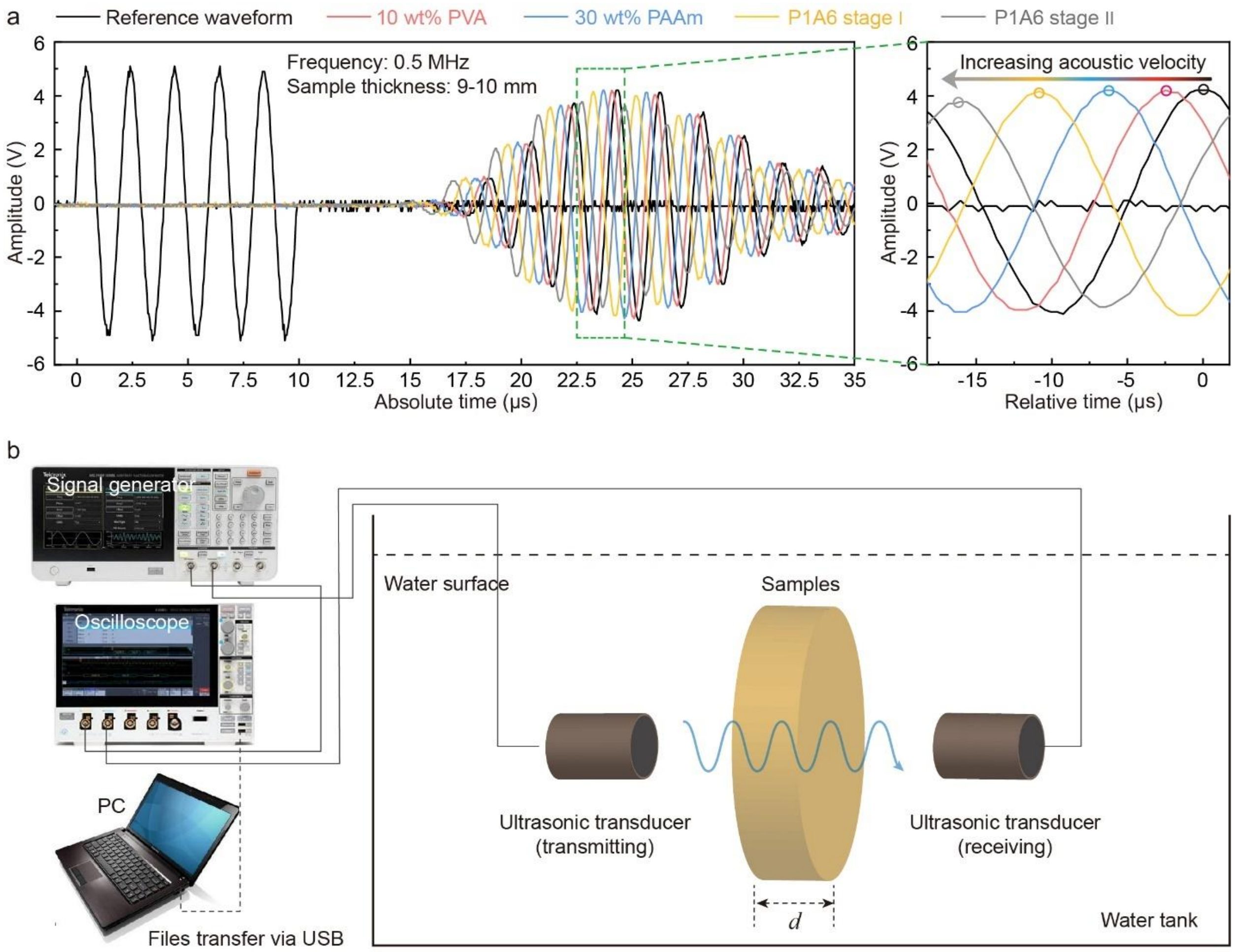

Figure S4. Experimental measurement of acoustic properties. (a) Sound speed measurements for pure water and hydrogel metamaterials (10wt% PVA hydrogel, 30wt% PAAm hydrogel, P1A6 hydrogel of stage I and stage II). The curves indicate that the ultrasound signals travel through the samples from the transmitting transducer to the receiving transducer with different source frequencies (0.5 MHz, 1 MHz, and 2 MHz). This diagram represents the ultrasound signal at 0.5 MHz frequency. The image on the right shows a zoomed-in view of the four hydrogels at their maximum amplitude, with the time zero point being the moment of maximum amplitude of the reference waveform (pure water). The four hydrogels exhibit increasing sound speed. (b) Experimental setup of acoustic measurement.

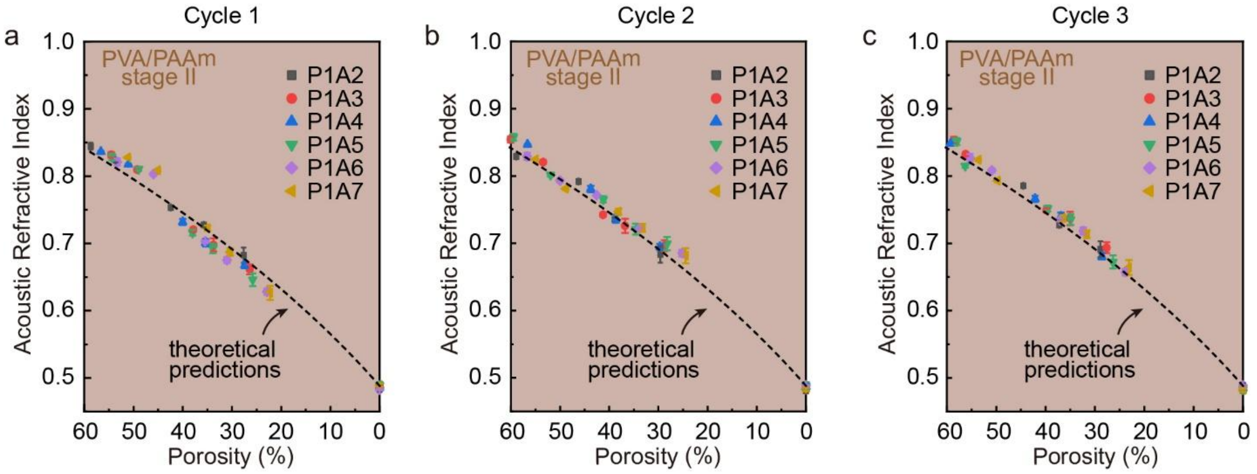

Figure S5. Experimental verification of the reversible porosity-refractive index relationship across multiple drying-rehydration cycles for the PVA/PAAm double-network hydrogels.

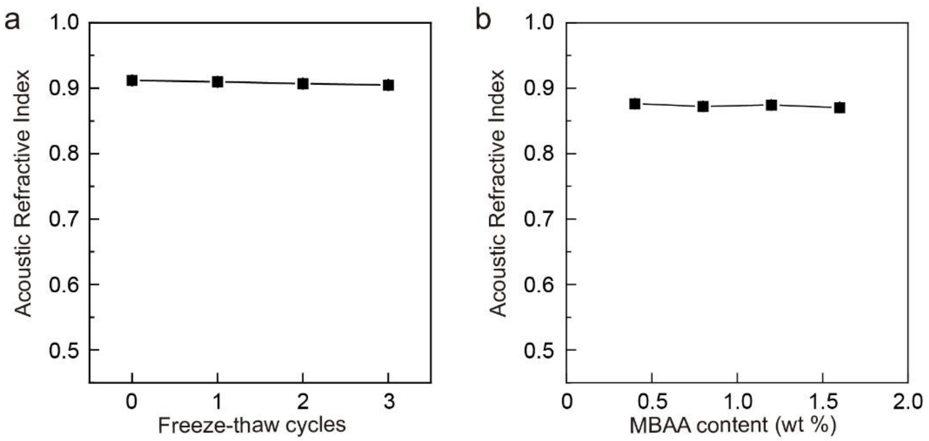

Figure S6. Effects of crystallinity and crosslink density on the acoustic refractive index of hydrogels. (a) Acoustic refractive index of the PVA/PAAm hydrogels as a function of the number of freeze-thaw cycles. Here, PVA/PAAm hydrogels refers to the crystallization of PVA by freezing and thawing instead of drying after the first stage. (b) Acoustic refractive index of PAAm hydrogels as a function of MBAA crosslinker mass fraction (relative to AAm monomer). Error bars represent three independent samples (n = 3), and error bars smaller than the symbol size are omitted.

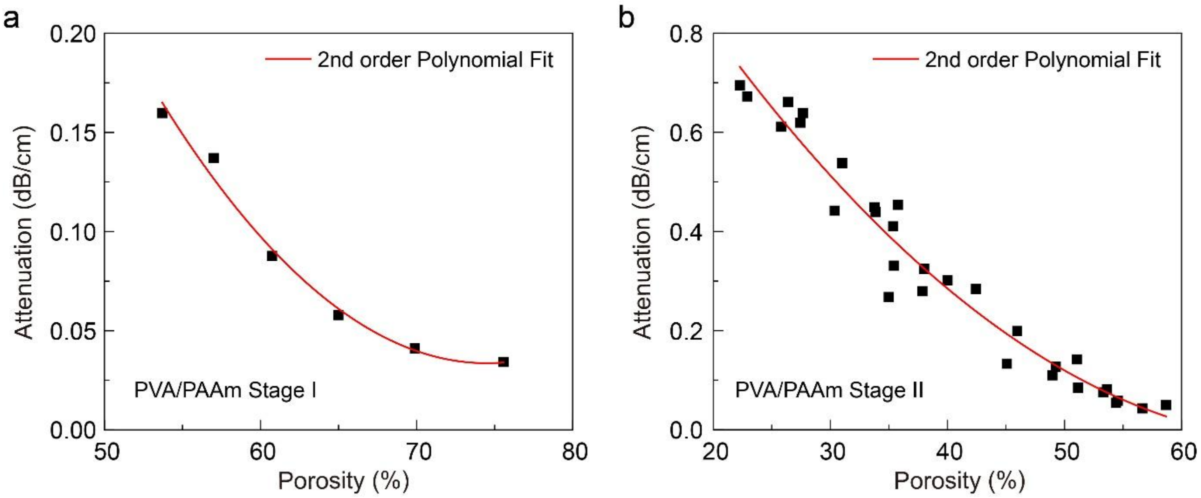

Figure S7. Statistical curve fitting of acoustic attenuation versus porosity for PVA/PAAm hydrogels. The plots display the experimental attenuation coefficients at 1 MHz (black squares) as a function of porosity for (a) Stage I and (b) Stage II hydrogels.

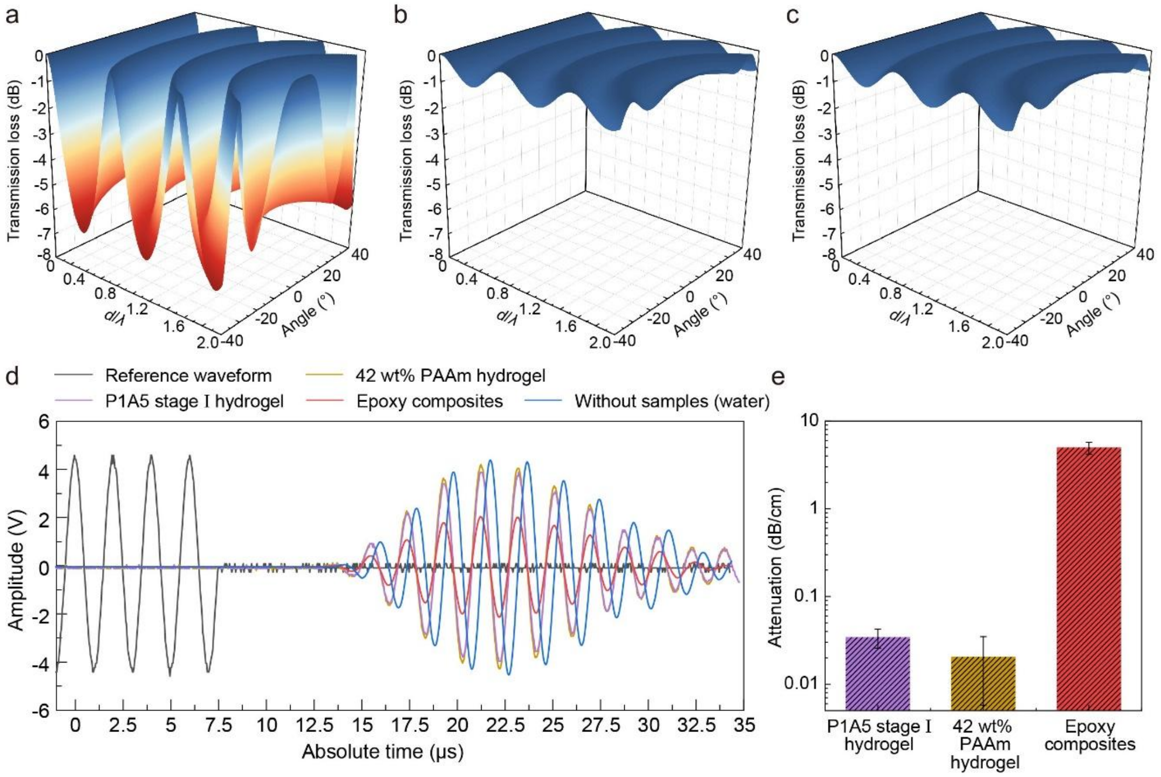

Figure S8. Evaluation of acoustic properties of hydrogel metamaterials and epoxy resin composite metamaterials. (a–c) Comparison of acoustic transmission through (a) the tungsten-epoxy composites, (b) 42 wt% PAAm hydrogels, and (c) P1A5 stage I hydrogels, respectively. The incident angle of the sound wave ranged from −40° to 40°. The horizontal coordinates represent the ratio of thickness d to wavelength λ. (d) The curves indicate that the ultrasound signals travel through the samples from the transmitting transducer to the receiving transducer at 0.5 MHz frequency. (e) Comparison of acoustic attenuation (at 0.5 MHz) among these three materials. Error bars represent standard deviation of three independent samples (n = 3).

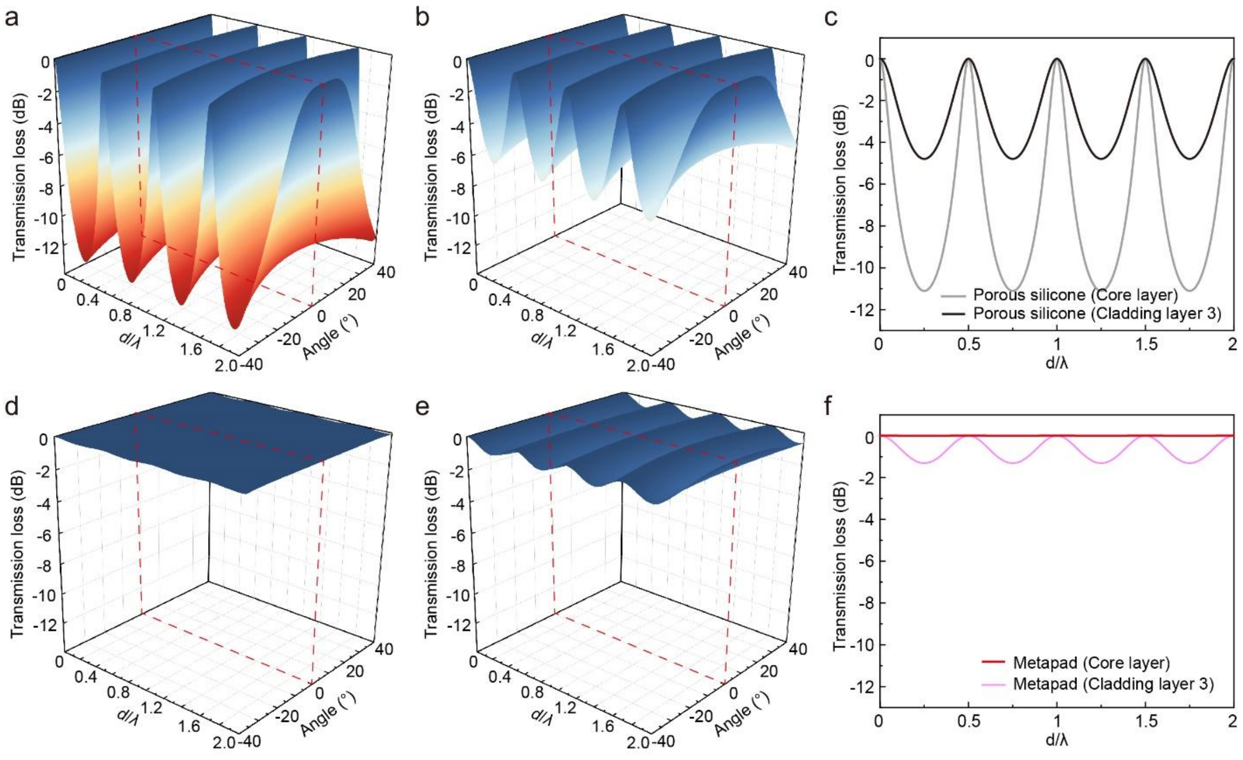

Figure S9. Evaluation of acoustic transmission properties of the metapad and porous silicone lens. (a–b) Acoustic transmission through (a) core layer and (b) cladding layer of the porous silicone lens. The incident angle of the sound wave ranged from −40° to 40°. The horizontal coordinates represent the ratio of thickness d to wavelength λ. (c) The transmission spectra of the two layers of porous silicone lens at normal incidence. The horizontal coordinates represent the ratio of thickness d to wavelength λ. (d–e) Acoustic transmission through (d) core layer and (e) cladding layer of the metapad. (f) The transmission spectra of the two layers of metapad at normal incidence.

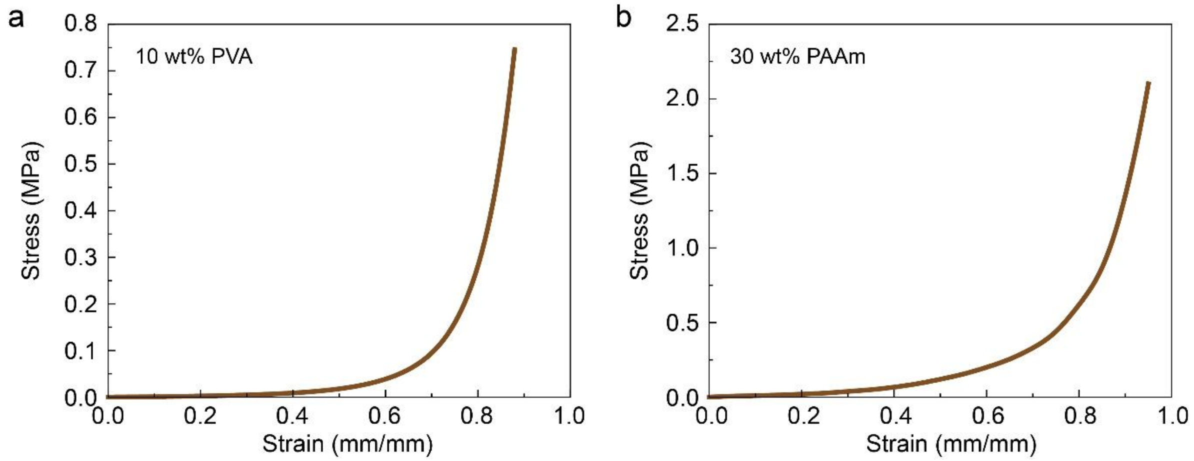

Figure S10. Compressive stress−strain curves of (a) the pure PVA and (b) pure PAAm hydrogels used in the metapad. The calculated compressive elastic modulus are 10.268 kPa and 100.169 kPa, respectively.

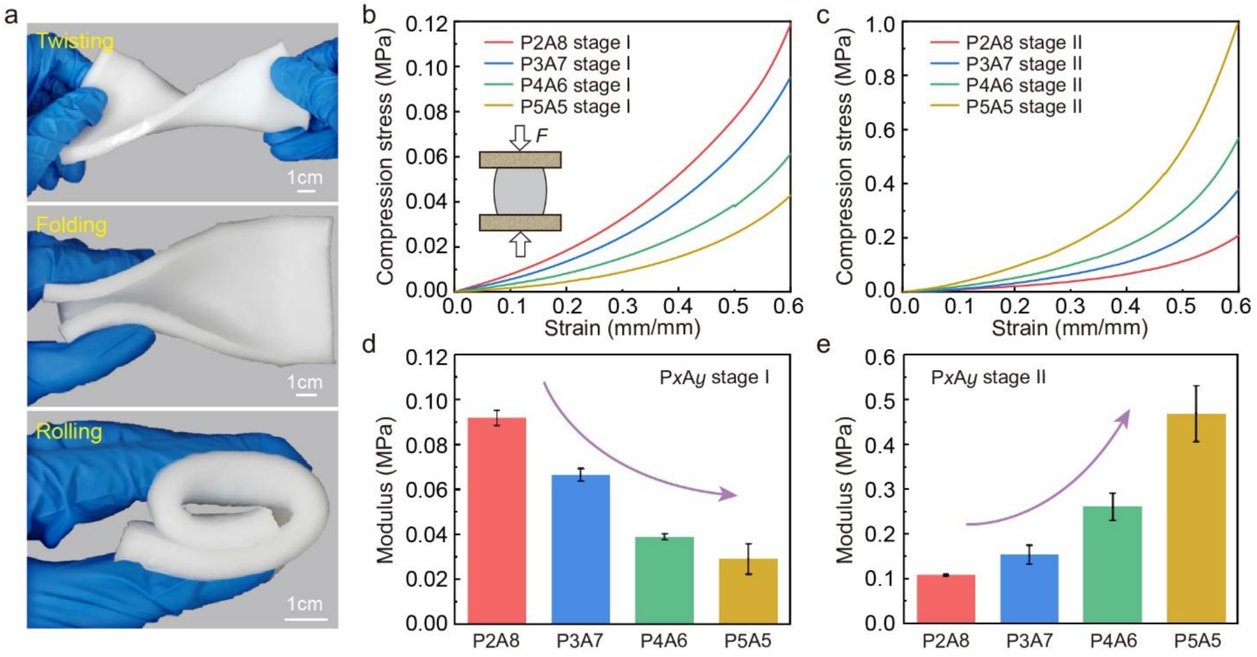

Figure S11. Mechanical performance of PVA/PAAm hydrogels constructing the metapad. (a) Images of twisting, folding, and rolling as-prepared hydrogel metamaterials (as an example of PVA/PAAm hydrogel of stage II) demonstrates its compliance, flexibility, and softness. (b–c) Compressive stress−strain curve of the PVA/PAAm hydrogel metamaterials of stage I and stage II. (d–e) Modulus of the PVA/PAAm hydrogel metamaterials of stage I and stage II.

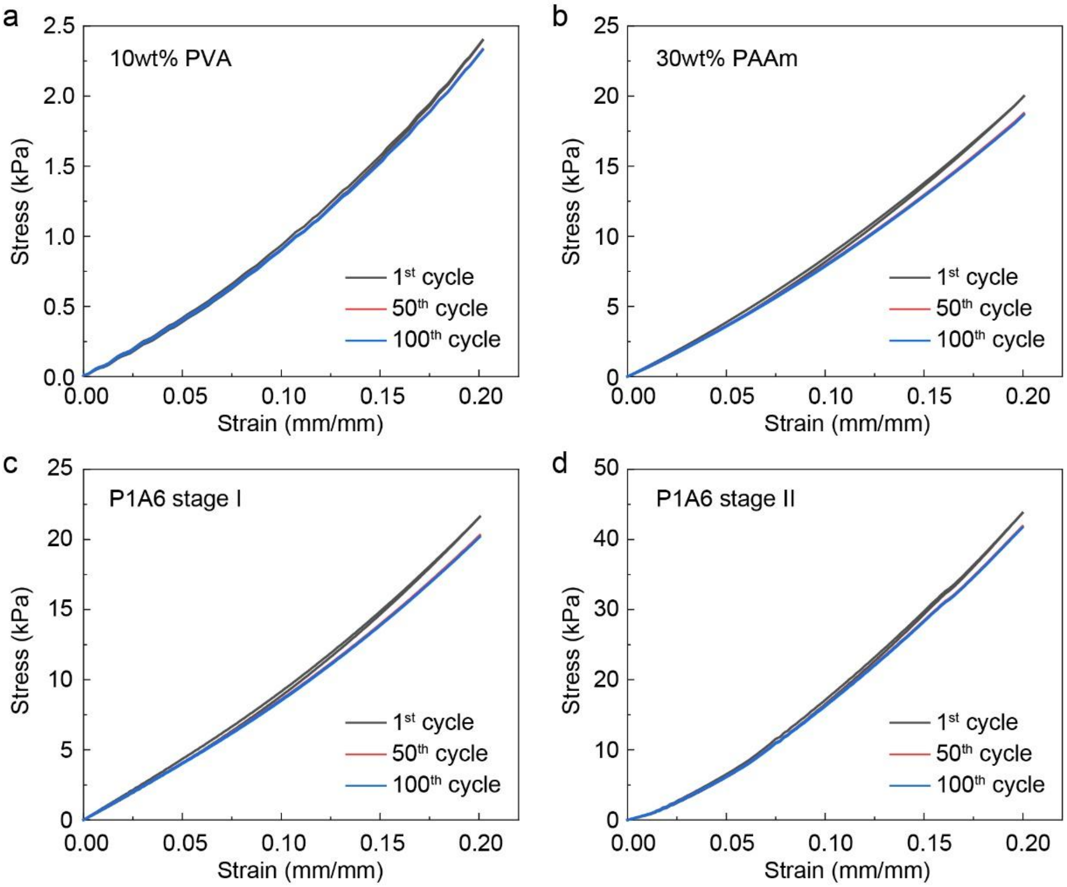

Figure S12. Mechanical durability characterization of the metapad constituent hydrogels under cyclic compression. Stress-strain curves of (a) 10wt% PVA, (b) 30wt% PAAm, (c) P1A6 stage I, and (d) P1A6 stage II hydrogels over 100 loading-unloading cycles.

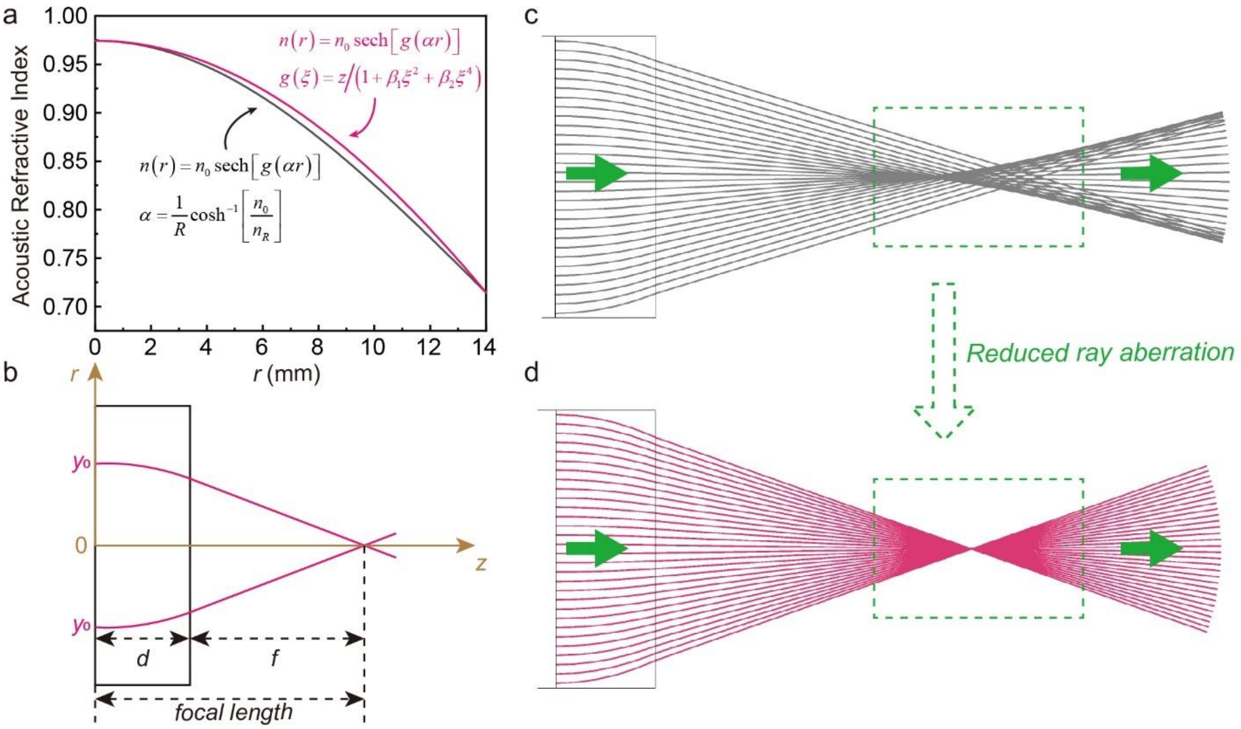

Figure S13. Acoustic refractive index profile of the hydrogel metapad. (a) Governing equations for conventional hyperbolic secant index profile (black curve) and modified hyperbolic secant index profile (red curve). (b) Schematic view of the metapad, along with two ray paths which focus a distance *f* from the metapad surface. (c–d) Ray trajectories for two metapads with conventional hyperbolic secant index profile and modified hyperbolic secant index profile, respectively.

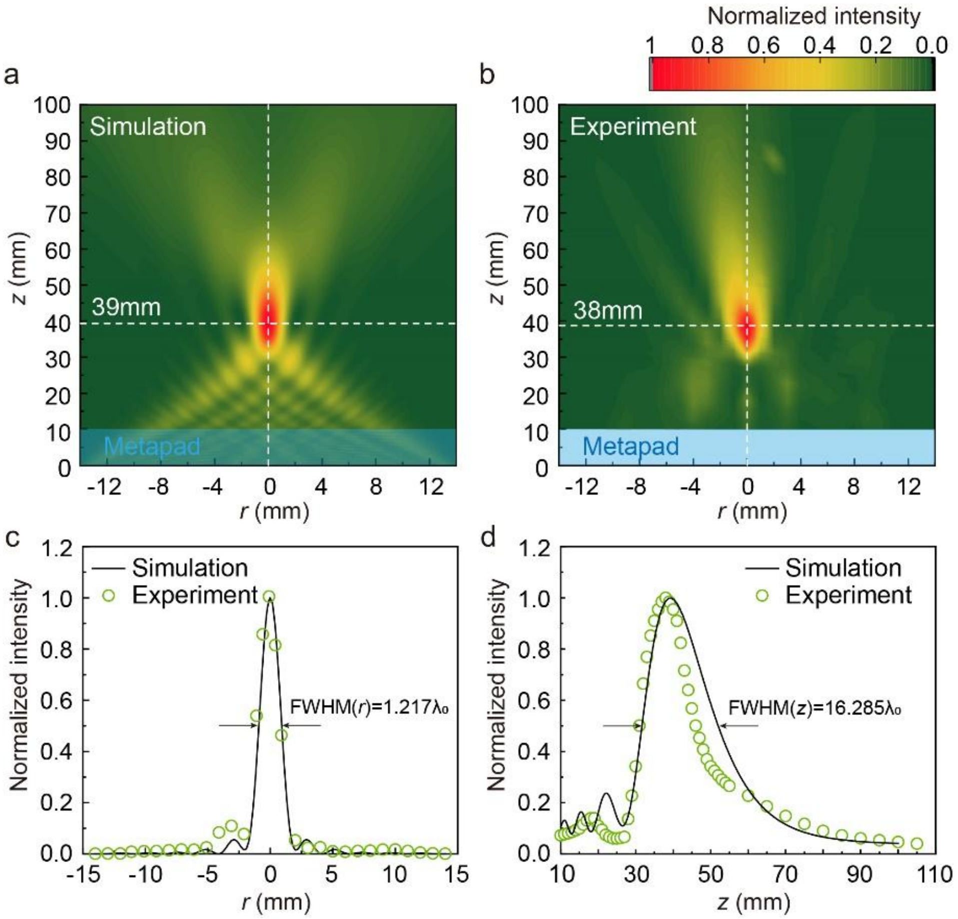

Figure S14. Ultrasound focusing capability of the metapad. (a–b) Acoustic intensity field patterns simulated and measured at 1 MHz in the *r*-*z* planes (28mm × 100mm) with the metapad deposited on a 28mm-diameter ultrasonic immersion transducer. The focal spot of the simulation is at z=39mm, and the focal spot of the experiment is at z=38mm. (c–d) Measured (green diamonds) and simulated (black line) normalized amplitude field distributions along the *z*-axis for r = 0 mm and along the *r*-axis for z = 39 mm and 38 mm. The full widths at half maximum peaks are $1.217\lambda_0$ and $16.285\lambda_0$ along the *r*-axis and *z*-axis, respectively.

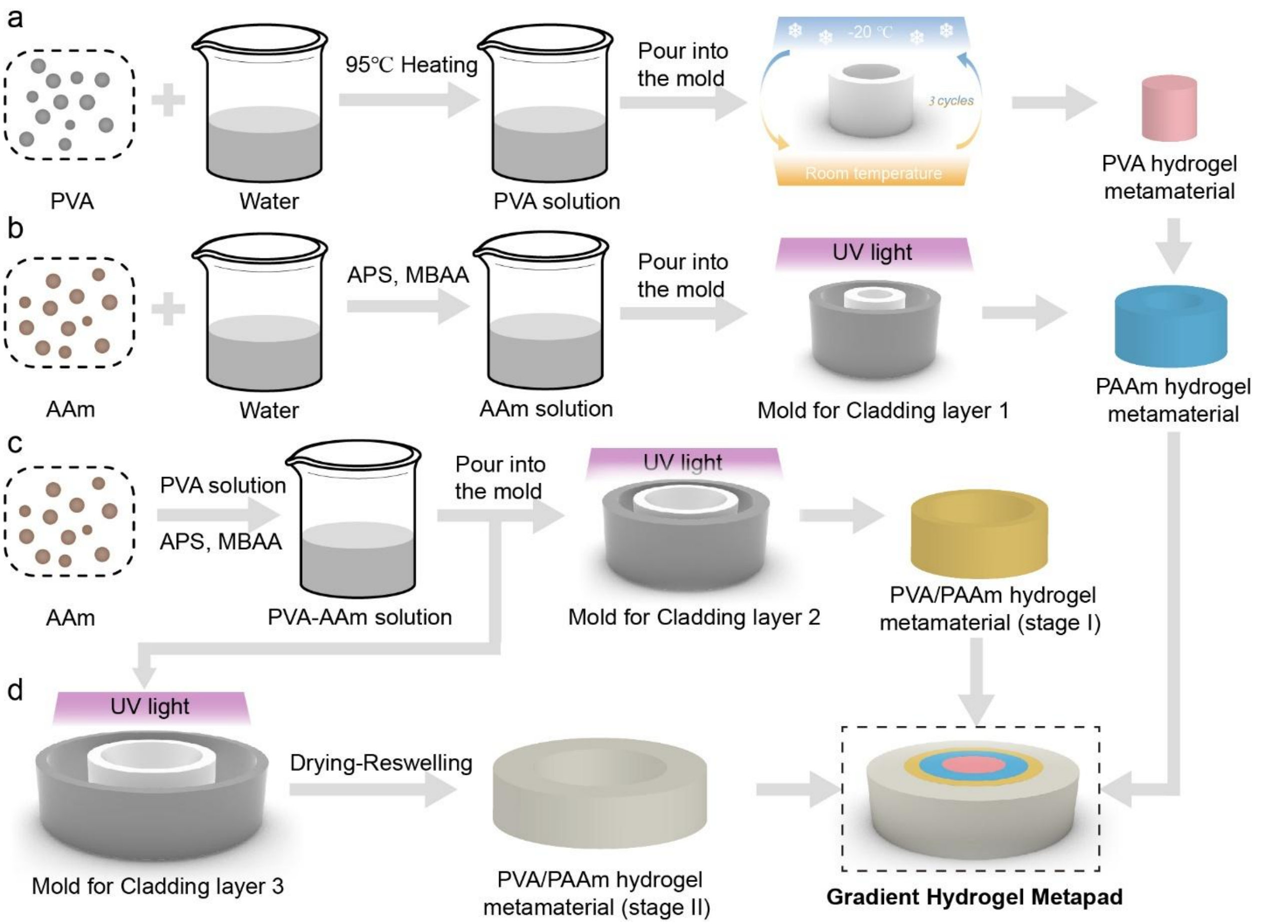

Figure S15. Fabrication and integration of the hydrogel metapad. (a) Fabrication process of the core layer (PVA hydrogel metamaterial) by cyclic freezing-thawing. (b) Fabrication process of the cladding layer 1 (PAAm hydrogel metamaterial) by chemical cross-linking. (c) Fabrication process of the cladding layer 2 (PVA/PAAm hydrogel metamaterial stage I). (d) Fabrication process of the cladding layer 3 (PVA/PAAm hydrogel metamaterial stage II) and integration process of the hydrogel metapad.

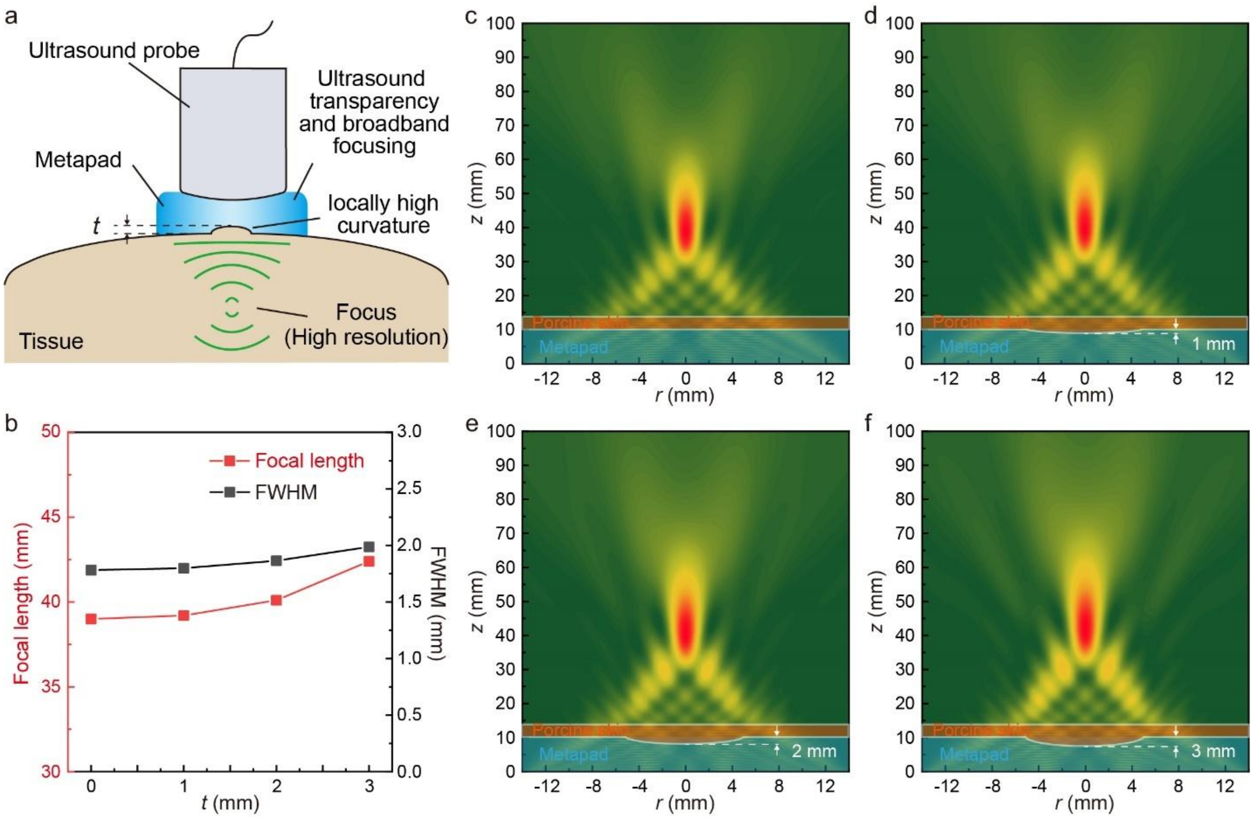

Figure S16. Ultrasound focusing capability of the hydrogel metapad through locally high curvature tissue surface. (a) Schematics of the hydrogel metapad attached to the tissue with locally high curvature. (b) Focal length and FWHM as a function of the thickness of the local high curvature tissue bulge. (c–f) Simulated ultrasound intensity distribution of 1 MHz ultrasound waves excited by the probe through different curvature tissue surfaces after hydrogel metapad manipulation.

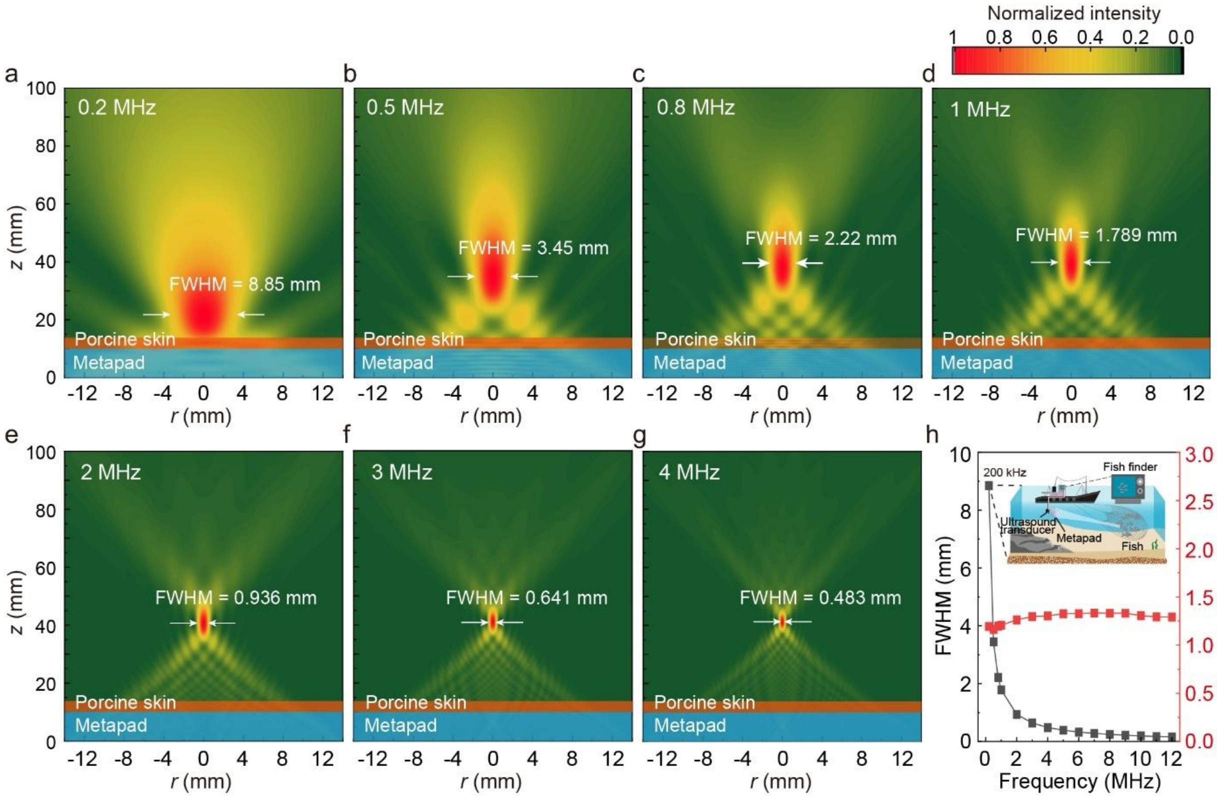

Figure S17. Broadband characteristics of the hydrogel metapad for ultrasound focusing. (a–g) Simulation of acoustic intensity distribution at 0.2 MHz, 0.5 MHz, 0.8 MHz, 1 MHz, 2 MHz, 3 MHz, and 4 MHz. The thickness of metapad is 1 cm. (h) Dependence of the FWHM on the operation frequency (from 0.2 MHz to 12 MHz). The metapad can also operate in bands commonly used in underwater acoustics, for example to improve fish finder beam characteristics and enhance fish detection.

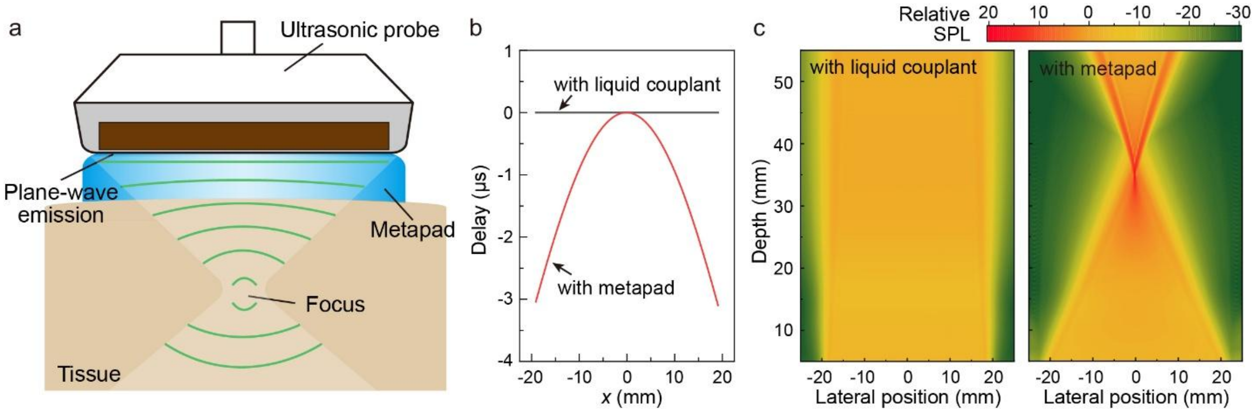

Figure S18. Simulations of ultrasound imaging via hydrogel metapad. (a) Schematic illustrating plane-wave imaging in the azimuthal plane via hydrogel metapad. (b) The delay of emitted acoustic waves from each element of the ultrasound probe arises due to the gradient acoustic refractive index present within the hydrogel metapad. (c) Field II simulation of the emitted field.

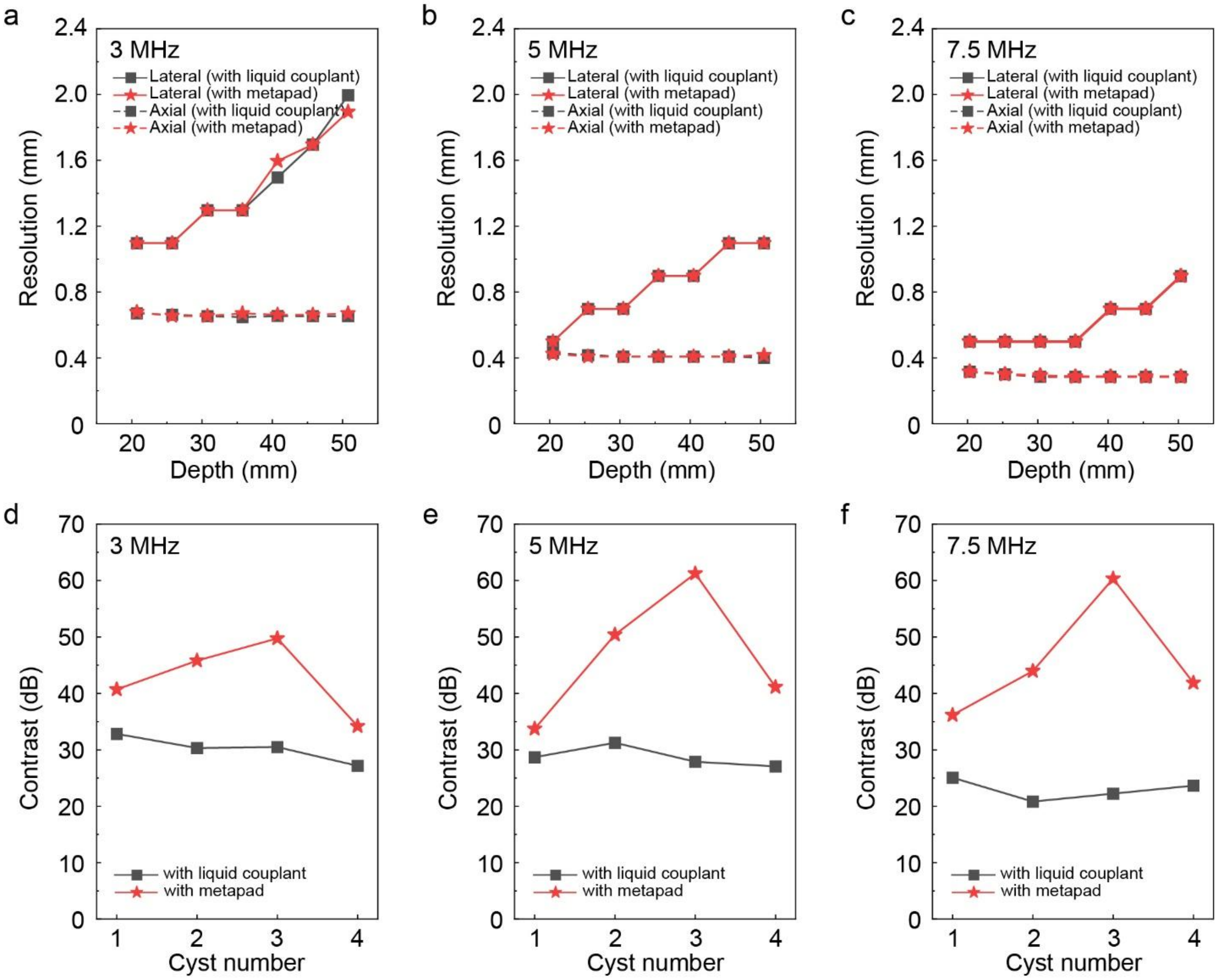

Figure S19. Broadband quantitative assessment of imaging resolution and contrast. (a–c) The −10 dB lateral and axial resolutions derived from the Point Spread Function (PSF) as a function of depth at excitation frequencies of 3 MHz, 5 MHz, and 7.5 MHz, respectively. (d–f) Quantitative contrast comparison of anechoic cysts at corresponding frequencies.

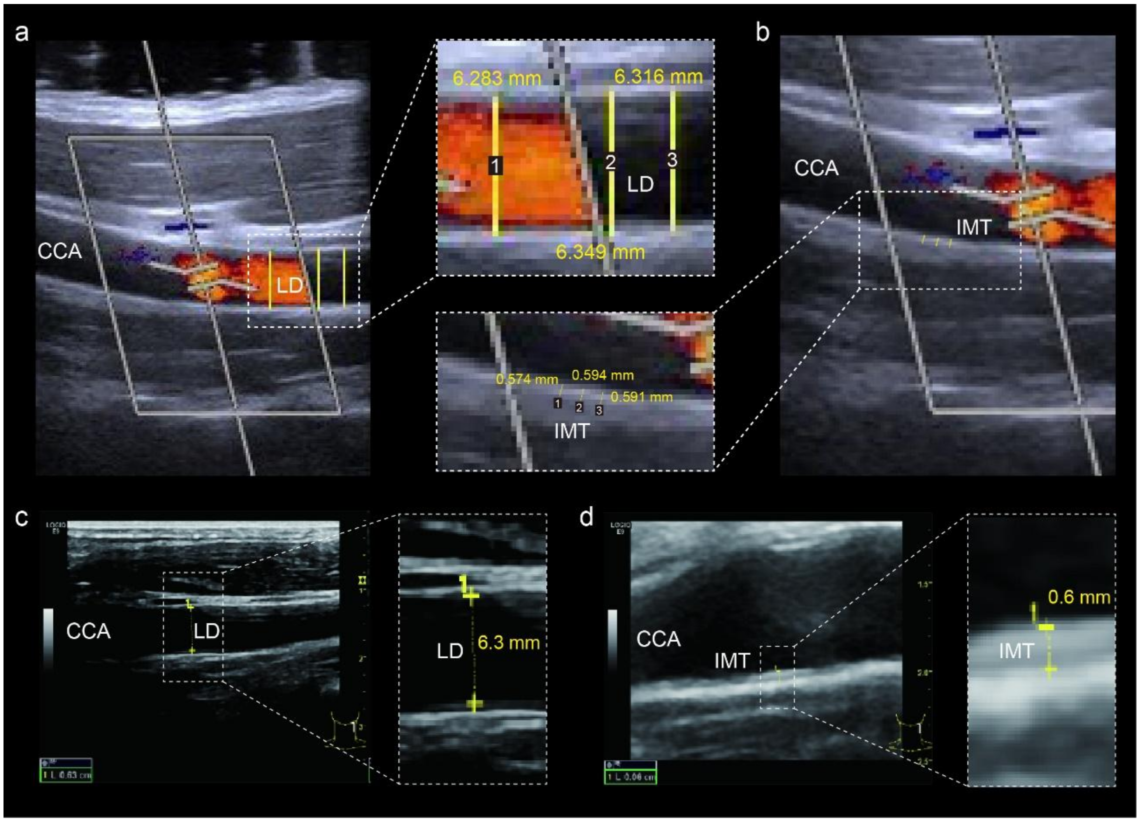

Figure S20. Ultrasonographic images and quantitative evaluation of the common carotid artery. (a–b) Ultrasound recording of the luminal diameter (LD) and intima-media thickness (IMT) using the metapad as the couplant. (c–d) The LD and IMT measurements using the commercial liquid couplant (TM-100; Jinya).

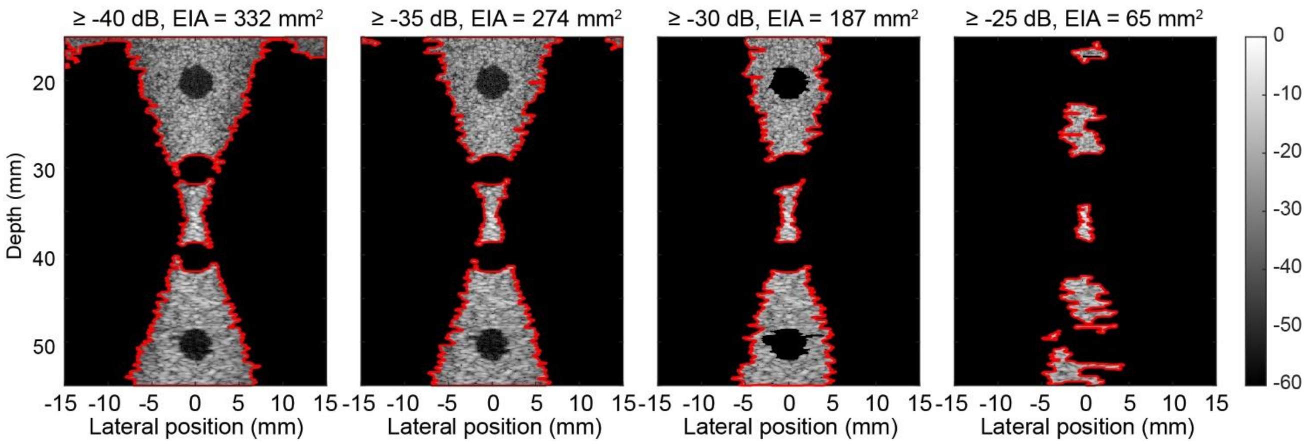

Figure S21. Quantitative Effective Imaging Area (EIA) for metapad-enhanced ultrasound imaging system.

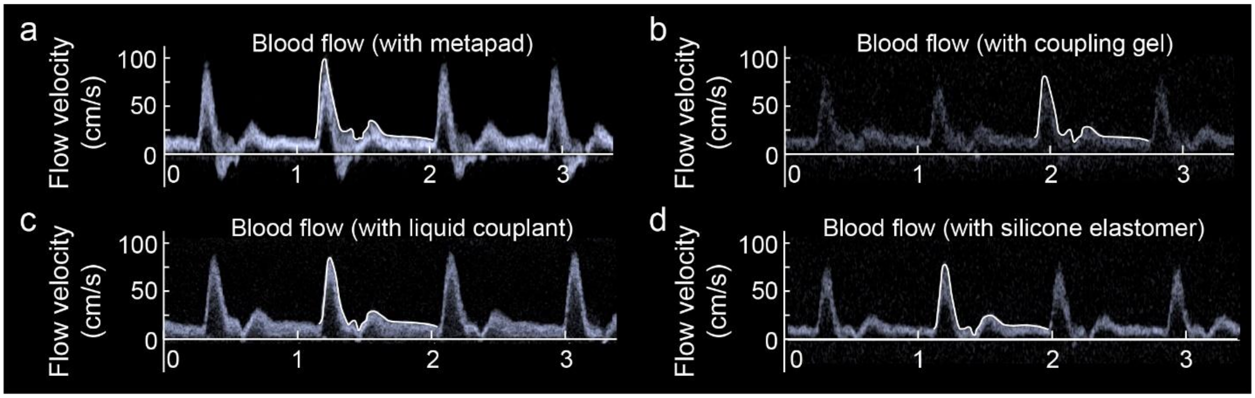

Figure S22. Blood flow in the carotid artery is imaged using (a) the metapad, (b) coupling gel, (c) liquid couplant, and (d) silicone elastomer.

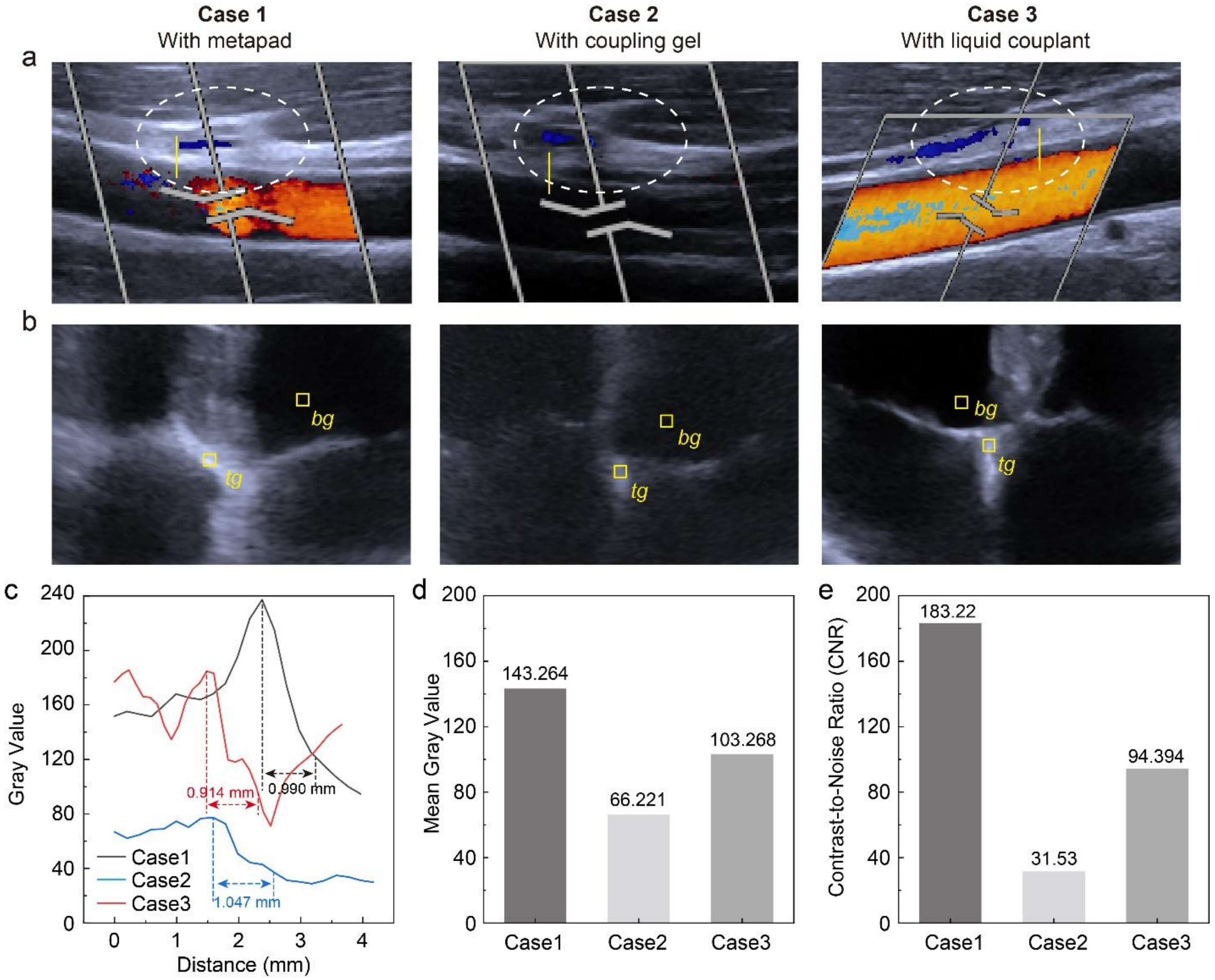

Figure S23. Quantitative evaluation of imaging performance. (a) Carotid artery images showing the measurement paths for resolution (yellow line) and the ROIs for signal intensity (white dashed box). (b) Heart images indicating the target and background ROIs (yellow squares) for CNR calculation. (c) Normalized axial intensity profiles across the vessel wall. (d) Comparative analysis of Mean Gray Value (MGV). (e) Comparative analysis of Contrast-to-Noise Ratio (CNR).

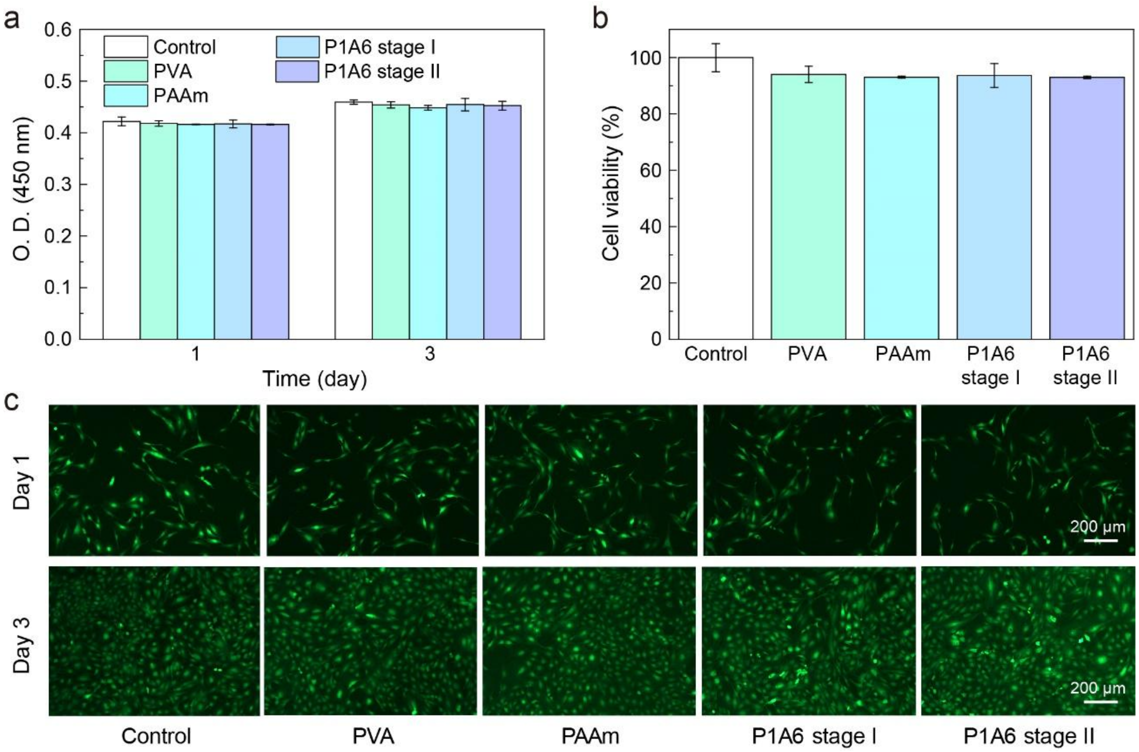

Figure S24. Cytotoxicity evaluation of the hydrogel metapad. (a) In vitro cell proliferation assays of hydrogels assessed by CCK-8 assay. (b) Quantitative analysis of data from the live staining assay (n = 3). (c) Fluorescence microscopy images of MC3T3-E1 cells after incubation with different hydrogels for 1 and 3 days (n = 3). Scale bars = 200 μm.

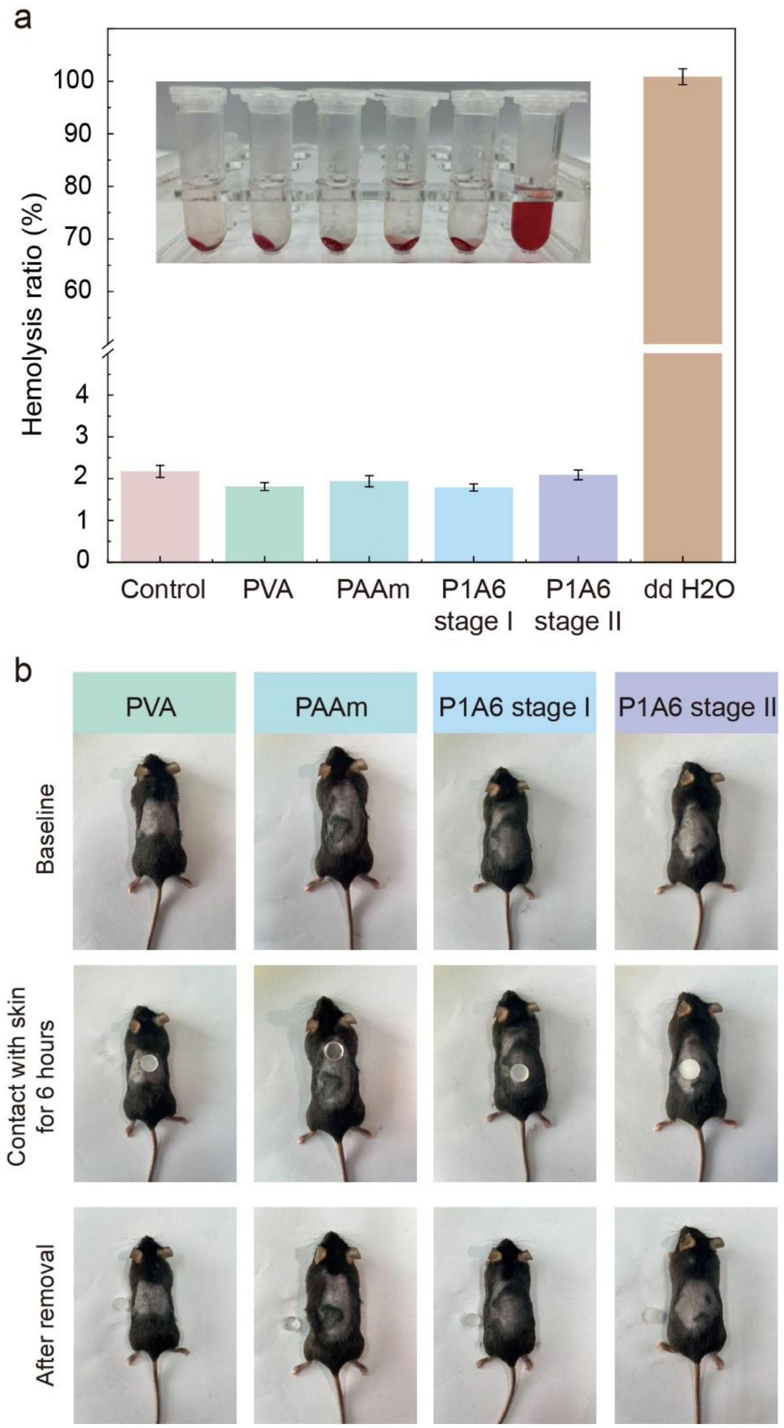

Figure S25. Blood and skin contact safety evaluation of the hydrogel metapad. (a) Blood compatibility assessed via a hemolysis test. Data are shown as mean ± standard deviation (n = 3). (b) Skin compatibility assessment. Representative photographs show the baseline skin condition, during 6 h of continuous contact, and after samples removal.

Table S1. Comparison of physical characteristics of the hydrogel metapad, ultrasound coupling devices, and acoustic functional device.

Types of coupling media	Softness	Ultrasound transparency	Ultrasound focusing	Biomedical ultrasound broadband	Gradient refractive index
Metapad (*This work*)	○	○	○	○	○
BAUS couplant [15]	○	○	×	○	×
Wearable imager [16]	○	○	×	○	×
Ultrasonic-system-on-patch, UsoP [17]	○	○	×	○	×
Doppler ultrasound patch [18]	○	○	×	○	×
Silk patch [19]	○	○	×	○	×
Bilayer hydrogel pad [20]	○	○	×	○	×
Double network hydrogel [21]	○	○	×	○	×
TM-100 (Jinya, liquid/gel couplant)	×	○	×	○	×
Ultrasound Gel Pad (Parker Aquaflex)	○	○	×	○	×
Acoustic metamaterial matching layer [22]	×	○	×	○	×
Porous silicone lens [23]	○	×	○	×	○
Bio-metamaterials lens [24]	○	×	○	×	○
Meta-skin lens [25]	○	×	○	×	×
Silicone Fresnel zone plate lens [26]	○	○	○	×	×
Phase advance lens [27]	×	×	○	×	○
Penta-mode lens [10]	×	×	○	×	○

Table S2. Comparison of acoustic properties of the metapad based on hydrogel metamaterials and common coupling media [15,21].

Types of coupling media	Sound speed (m/s)	Acoustic impedance (MRayl)	Attenuation coefficient at 2MHz (dB/cm)	Sound transmission coefficient	Sound focusing efficiency
Metapad (*This work*)	1500–2000	1.5–2.6	0.1–0.8	0.963–0.994	5.175 dB (at 1 MHz)
Hydrogel-elastomer hybrid (BAUS couplant)	1548	1.59	0.256	0.997	/
Liquid hydrogel (Aquasonic Clear, Parker Labs)	1538	1.61	0.598	0.998	/
Solid hydrogel (Aquaflex, Parker Labs)	1595	1.65	1.068	0.999	/
Polyacrylamide/Alginate hydrogel	1511–1600	1.53–1.65	0.26–0.5	0.995–0.999	/
Elastomer couplants (Ecoflex, Smooth-On)	989	1.06	1.966	0.938	/
Aqualene (Olympus)	1586	1.46	2.436	0.991	/
Polyurethane (Sigma-Aldrich)	1040	1.59	6.239	0.997	/
Acrylic tape (VHB, 3M)	1520	1.49	12.906	0.993	/
Ultrasound Gel Pad (Parker Aquaflex)	1595	1.65	1.025	0.999	/

*Slash (/) indicates no focusing capability.

Table S3. Comparison of the hydrogel metapad and existing acoustic focusing lenses.

Types of acoustic focusing lens	Soft or stiff	Operation frequency (MHz)	Acoustic impedance (MRayl)	Attenuation coefficient of building materials (dB/cm)	Ultrasonic transparent	Broadband focusing	Practical application of biomedical ultrasound
Hydrogel Metapad	Soft	0.05–4 (Broadband)	1.5 (Core layer) 2.6 (Outermost layer)	0.02 (Core layer, 1 MHz) 0.38 (Outermost layer, 1 MHz)	○	○	Ultrasound imaging of human tissues
Porous silicone lens [23]	Soft	0.09–0.2 (Broadband)	0.21 (Core layer) 0.47 (Outermost layer)	96.20 (Core layer, 0.2 MHz) 34.45 (Outermost layer, 0.2 MHz)	×	○	/
Bio-metamaterials lens [24]	Soft	0.06–0.12 (Broadband)	1.665 (Core layer) 1.087 (Outermost layer)	32.82 (Core layer, 1 MHz) 1.216 (Outermost layer, 1 MHz)	×	○	/
Meta-skin lens [25]	Soft	5 (Single frequency)	0.415E−3 (Meta-skin ring) 1.483 (Water ring)	/	×	×	/
Silicone Fresnel zone plate lens [26]	Soft	1 (Single frequency)	1.24	/	○	×	Bone quality assessment
Phase advance lens [27]	Rigid	0.07–0.025 (Broadband)	/	/	×	○	/
Penta-mode lens [10]	Rigid	0.015–0.04 (Broadband)	/	/	×	○	/

Table S4. Statistical validation of theoretical predictions and empirical fits for acoustic properties.

Physical Property	Material System	Analysis Method / Model	Coefficient of Determination (R^2)
Acoustic Refractive Index (n)	PVA hydrogels	Theory Validation<br>(Waterman-Truell Model, Eq. 4)	0.99999
	PAAm hydrogels	Theory Validation<br>(Waterman-Truell Model, Eq. 4)	0.99968
	PVA/PAAm hydrogels<br>(Stage I)	Theory Validation<br>(Waterman-Truell Model, Eq. 4)	0.99568
	PVA/PAAm DN hydrogels<br>(Stage II)	Theory Validation<br>(Waterman-Truell Model, Eq. 4)	0.99961
Attenuation (α)<br><br>(at 1 MHz)	PVA/PAAm hydrogels<br>(Stage I)	Empirical Regression<br>(Polynomial Fit, Order 2)<br>Equation: $\alpha = A + B\varphi + C\varphi^2$<br>Parameters: A=1.72005, B=−0.04527, C= 3.03859E−4	0.98832
	PVA/PAAm DN hydrogels<br>(Stage II)	Empirical Regression<br>(Polynomial Fit, Order 2)<br>Equation: $\alpha = A + B\varphi + C\varphi^2$<br>Parameters: A=1.57258, B=−0.04472, C= 3.13138E−4	0.96042

Table S5. Acoustic impedance and attenuation coefficients at 0.5, 1, and 2 MHz of the four hydrogels used in the metapad design.

Hydrogel type	0.5 MHz		1 MHz		2 MHz	
	Impedance (MRayl)	Attenuation (dB/cm)	Impedance (MRayl)	Attenuation (dB/cm)	Impedance (MRayl)	Attenuation (dB/cm)
10 wt% PVA	1.632	0.07921	1.631	0.09138	1.631	0.11191
30 wt% PAAm	1.909	0.01164	1.900	0.05311	1.896	0.07201
P1A6 stage I	2.068	0.04027	2.070	0.13702	2.071	0.17833
P1A6 stage II	2.705	0.15324	2.710	0.33094	2.713	0.48239

Table S6. Parameters of ultrasound transducer combined with metapad for ultrasound imaging [28].

Type of ultrasound transducer	10L4 (ACUSON Sequoia)	4V1 (ACUSON Sequoia)
Number of elements	384	128
Bandwidth	2.9–9.9 MHz	1.4–5.1 MHz
Selectable frequencies	Low, Mid, High*	Low, Mid*, High
Axial & lateral resolution	0.3 & 0.52 mm	0.9 & 1.1 mm
Physical footprint	49.25 × 18.85 mm	35.5 × 20.2 mm

Note: Asterisk (*) indicates the frequency options used in the ultrasound imaging demonstration.

Table S7. Acoustic parameters of the discretized metapad for ultrasound imaging.

Layers	Position (mm)	Types of hydrogel	Sound velocity (m/s)	Refractive index	Density (kg/m^3)	Acoustic impedance ($\times 10^6$ $N \cdot s/m^3$)
Core layer	0–5	PVA hydrogel (12.5 wt%)	1522	0.974	1027.1	1.56
Cladding layer 1	5–10	PAAm hydrogel (20 wt%)	1570	0.945	1016.9	1.60
Cladding layer 2	10–15	PAAm hydrogel (28 wt%)	1640	0.904	1024.6	1.68
Cladding layer 3	15–20	P1A5 stage 1 hydrogel	1750	0.847	1056.3	1.85
Cladding layer 4	20–25	P1A5 stage 2 hydrogel	1930	0.768	1171.9	2.26

Table S8. Quantitative comparison of technical specifications between the hydrogel metapad and commercial and flexible arrays.

Technical Metric	Commercial Active Array	Flexible/Stretchable Array	Hydrogel Metapad (This Work)
Element Pitch (d)	~ 0.2 mm<br>(*Fixed by manufacturing*)	0.4 mm [16]<br>0.3 mm [15]<br>(*Limited by wiring/stretchability*)	$d \to 0$<br>(*Based on continuous gradient medium*)
Grating Lobe Threshold ($f_{max} \approx c/d$ for vertical focusing [29])	~ 7.7 MHz<br>(*Based on d = 0.2 mm*)	~ 5.13 MHz [16]<br>~ 3.85 MHz [15]	None observed
Grating Lobe Artifacts (at High Freq, e.g., 9 MHz)	Lobes appear at $\theta = \arcsin(\lambda/d)$ [29]	At 9 MHz, $d > 2\lambda$, causing strong aliasing and multiple false foci.	No spatial aliasing due to gradient index
Mainlobe-to-Sidelobe Ratio (MSR)	Rapidly degrades if aliased		Not limited by aliasing
Point Spread Function (PSF)	The minimum achievable width of the PSF is determined by the diffraction limit. As the frequency increases, the main-lobe width of the PSF—corresponding to the lateral resolution—progressively improves.		
Resolution (Lateral)	Typical value: ~ 1.0–1.5λ<br>(at central frequency)	1.55 mm at frequency of 3 MHz and a depth of 3 cm [16].<br><br>1.79 mm at frequency of 3 MHz and a depth of 30 mm [15].	0.50 mm at frequency of 7.5 MHz and a depth of 3.5 cm.
Contrast	Relatively flat/uniform profile		Shows a significant peak near the focal region

Table S9. Parameters used for ultrasound imaging simulation.

Parameter	Transducer
Number of elements	192
Center frequency, f_0	7.5 MHz
Sound speed of tissue, c	1540
Attenuation, α	0.5 dB/cm/MHz
Wavelength, λ	0.205 mm
Sampling frequency	100 MHz
Element pitch	0.2 mm
Element height	5 mm
Cycles m in emitted pulse	1
Transmit apodization	Hanning
Receive apodization	Hanning

Supplementary References

1. Ping He. Experimental verification of models for determining dispersion from attenuation. *IEEE Trans Ultrason Ferroelectr Freq Control* 1999; **46**: 706–14.

2. Kim HY, Gutierrez B, Nelson T *et al. Using the acoustic Doppler current profiler (ADCP) to estimate suspended sediment concentration*. University of South Carolina: Columbia, SC, USA, 2004: 04–01.

3. Chen H, Zhao Z, Zhang R *et al.* Adaptable hydrogel with strong adhesion of wet tissue for long-term protection of periodontitis wound. *Adv Mater* 2025; **37**: 2413373.

4. Kinsler LE, Frey AR, Coppens AB *et al. Fundamentals of Acoustics*. John Wiley & Sons, 2000.

5. Parker KJ, Lerner RM, Waag RC. Attenuation of ultrasound: magnitude and frequency dependence for tissue characterization. *Radiology* 1984; **153**: 785–8.

6. Montaldo G, Tanter M, Bercoff J *et al.* Coherent plane-wave compounding for very high frame rate ultrasonography and transient elastography. *IEEE Trans Ultrason Ferroelectr Freq Control* 2009; **56**: 489–506.

7. Tiran E, Deffieux T, Correia M *et al.* Multiplane wave imaging increases signal-to-noise ratio in ultrafast ultrasound imaging. *Phys Med Biol* 2015; **60**: 8549.

8. Fu Y, Kabir II, Yeoh GH *et al.* A review on polymer-based materials for underwater sound absorption. *Polym Test* 2021; **96**: 107115.

9. Mikaelian AL, Prokhorov AM. Self-focusing media with Variable Index of Refraction. In: Wolf E (ed.). *Progress in Optics*. Elsevier, 1980, 279–345.

10. Su X, Norris AN, Cushing CW *et al.* Broadband focusing of underwater sound using a transparent pentamode lens. *J Acoust Soc Am* 2017; **141**: 4408–17.

11. Dmochowski J, Benesty J, Affes S. On Spatial Aliasing in Microphone Arrays. *IEEE Trans Signal Process* 2009; **57**: 1383–95.

12. Koskinen SM, Soinne L, Valanne L *et al.* The normal internal carotid artery: a computed tomography angiographic study. *Neuroradiology* 2014; **56**: 723–9.

13. De Groot E, Van Leuven SI, Duivenvoorden R *et al.* Measurement of carotid intima–media thickness to assess progression and regression of atherosclerosis. *Nat Clin Pract Cardiovasc Med* 2008; **5**: 280–8.

14. Bushberg JT, Seibert JA, Leidholdt EM *et al. The Essential Physics of Medical Imaging*. Lippincott Williams & Wilkins, 2011.

15. Wang C, Chen X, Wang L *et al.* Bioadhesive ultrasound for long-term continuous imaging of diverse organs. *Science* 2022; **377**: 517–23.

16. Hu H, Huang H, Li M *et al.* A wearable cardiac ultrasound imager. *Nature* 2023; **613**: 667–75.

17. Lin M, Zhang Z, Gao X *et al.* A fully integrated wearable ultrasound system to monitor deep tissues in moving subjects. *Nat Biotechnol* 2024; **42**: 448–57.

18. Kenny J-ÉS, Munding CE, Eibl JK *et al.* A novel, hands-free ultrasound patch for continuous monitoring of quantitative Doppler in the carotid artery. *Sci Rep* 2021; **11**: 7780.

19. Lee S-M, Lee T, Kim H *et al.* Calcium-Modified Silk Patch as a Next-Generation Ultrasound Coupling Medium. *ACS Appl Mater Interfaces* 2021; **13**: 55827–39.

20. Chen L, Zeng G, Guo D *et al.* Soft elastic hydrogel couplants for ultrasonography. *Mater Sci Eng C* 2021; **119**: 111609.

21. Yi J, Nguyen K-CT, Wang W *et al.* Polyacrylamide/Alginate double-network tough hydrogels for intraoral ultrasound imaging. *J Colloid Interface Sci* 2020; **578**: 598–607.

22. Li Z, Yang D-Q, Liu S-L *et al.* Broadband gradient impedance matching using an acoustic metamaterial for ultrasonic transducers. *Sci Rep* 2017; **7**: 42863.

23. Jin Y, Kumar R, Poncelet O *et al.* Flat acoustics with soft gradient-index metasurfaces. *Nat Commun* 2019; **10**: 143.

24. Zhang J, Zhou N, Dong E *et al.* Soft bio-metamaterials with high acoustic transparency and gradient refractive index for tunable acoustic beamformer. *Matter* 2024; **7**: 3857–75.

25. Li Z-L, Chen K, Li F *et al.* Decorated bacteria-cellulose ultrasonic metasurface. *Nat Commun* 2023; **14**: 5319.

26. He J, Jiang X, Zhang C *et al.* Stretchable Ultrasound Metalens for Biomedical Zoom Imaging and Bone Quality Assessment with Subwavelength Resolution. *Small* 2024; **20**: 2312221.

27. Martin TP, Naify CJ, Skerritt EA *et al.* Transparent Gradient-Index Lens for Underwater Sound Based on Phase Advance. *Phys Rev Appl* 2015; **4**: 034003.

28. ACUSON Ultrasound Machines. https://www.siemens-healthineers.com/ultrasound.

29. Szabo TL. *Diagnostic Ultrasound Imaging: Inside Out*. Academic Press, 2004.
